# Comprehensive Genome-Wide Survey, Genomic Constitution and Expression Profiling of the NAC Transcription Factor Family in Foxtail Millet (*Setaria italica* L.)

**DOI:** 10.1371/journal.pone.0064594

**Published:** 2013-05-15

**Authors:** Swati Puranik, Pranav Pankaj Sahu, Sambhu Nath Mandal, Venkata Suresh B., Swarup Kumar Parida, Manoj Prasad

**Affiliations:** National Institute of Plant Genome Research, Aruna Asaf Ali Marg, New Delhi, India; Nanjing Agricultural University, China

## Abstract

The NAC proteins represent a major plant-specific transcription factor family that has established enormously diverse roles in various plant processes. Aided by the availability of complete genomes, several members of this family have been identified in *Arabidopsis*, rice, soybean and poplar. However, no comprehensive investigation has been presented for the recently sequenced, naturally stress tolerant crop, *Setaria italica* (foxtail millet) that is famed as a model crop for bioenergy research. In this study, we identified 147 putative NAC domain-encoding genes from foxtail millet by systematic sequence analysis and physically mapped them onto nine chromosomes. Genomic organization suggested that inter-chromosomal duplications may have been responsible for expansion of this gene family in foxtail millet. Phylogenetically, they were arranged into 11 distinct sub-families (I-XI), with duplicated genes fitting into one cluster and possessing conserved motif compositions. Comparative mapping with other grass species revealed some orthologous relationships and chromosomal rearrangements including duplication, inversion and deletion of genes. The evolutionary significance as duplication and divergence of NAC genes based on their amino acid substitution rates was understood. Expression profiling against various stresses and phytohormones provides novel insights into specific and/or overlapping expression patterns of *SiNAC* genes, which may be responsible for functional divergence among individual members in this crop. Further, we performed structure modeling and molecular simulation of a stress-responsive protein, SiNAC128, proffering an initial framework for understanding its molecular function. Taken together, this genome-wide identification and expression profiling unlocks new avenues for systematic functional analysis of novel *NAC* gene family candidates which may be applied for improvising stress adaption in plants.

## Introduction

Agricultural productivity and yields are dependent upon the environment a crop encounters during its growth. In order to burgeon, a species must learn adaptive approaches against these recurrent challenges. Grasses belonging to the genus *Setaria* which have a world-wide existence, provide a fine example of such species. Particularly, *Setaria italica*, which was domesticated from the problematic weed *Setaria viridis* >8700 years ago, is amongst the oldest cultivated crops. This abiotic stress-tolerant grass has presented itself as an ideal model for understanding biological processes in potential biofuel grasses such as switchgrass, napier grass and pearl millet as they have closely-related but comparatively complex genome [1]. Considering this importance, its genome has been recently sequenced by two independent groups viz., Joint Genome Institute, Department of Energy, USA and Beijing Genomics Institute, China [1], [2].

Preliminary analysis of the draft genome has revealed that the stress-adaptive characteristics which foxtail millet possesses have yet not been evolved in other plants. Stress adaptation is a complex incident as stress may occur at diverse stages of plant development and often multiple stresses concurrently affect the plant [3]. Comprehensive investigations have revealed the molecular stress adaptation mechanisms which are governed by processes that allow regulated gene expression by an accurate signaling and tight transcriptional control. This entails binding of specific transcription factors (TFs) to *cis*-regulatory sequences in promoter of a stress-responsive gene. A corollary of this fact is that plants donate a large part of their genome (?7%) to encode TFs belonging several families, such as AP2/ERF, bZIP, NAC, MYB, MYC, Cys2His2, zinc-finger and WRKY, each with a dedicated binding site through which they can activate or repress the expression of their respective target genes [4].

As a crucial form of TFs, the well-known *NAC* gene family has emerged as a complex plant-specific superfamily. The ellipsis, NAC, derives its name from three earliest characterized proteins from petunia NAM (no apical meristem), *Arabidopsis* ATAF1/2 and CUC2 (cup-shaped cotyledon) [5]. This family has been noted for the presence of numerous members in the model plant Arabidopsis (*Arabidopsis thaliana*: 117), crops such as rice (*Oryza sativa*: 151), soybean (*Glycine max*: 152), tree species like poplar (*Populus trichocarpa*: 163), grape (*Vitis vinifera*: 79) and tobacco (*Nicotiana tabacum*: 152) [6]–[11]. It is characterized by the presence of a highly conserved NAC domain at N-terminal of the protein, however some exceptions have also been noted [5]. Although this domain confines the ability of DNA binding (DB), it shows great variation in recognizing DB sites in the target genes and atleast 5 different arrays have been identified [5], [10]–[19]. This feature allows them to regulate spatial and temporal expression of a variety of downstream genes towards governing multiple cellular or molecular processes. The highly diverged C-terminal end functions as a transcription regulatory region, by conferring either activation [14], [20]–[22] or repression activity [15], [23], [24]. In some NAC proteins, these N- or C-terminal domains may modulate protein-protein interactions [5]. As an additional feature, some NAC proteins comprise a α-helical transmembrane (TM) motif for anchoring to plasma membrane or endoplasmic reticulum.

Genes encoding NAC proteins can be regulated (i) transcriptionally by upstream TFs such as ABREs (ABA-responsive elements) and DREs (Dehydration-responsive elements), (ii) post-transcriptionally by micro-RNAs or alternative splicing, and (iii) post-translationally by ubiquitinization, dimerization, phosphorylation or proteolysis [5]. These regulatory steps assists the functional involvement of NAC proteins in majority of plant processes including orchestration of organ, fiber and secondary wall development [18], [25]–[27], cell cycle control [28]–[30], and senescence [31],[32]. Their multi-functionality has also been implicated in the regulation of molecular pathways that govern abiotic and biotic stress responses through mediation by hormones [5], [25], [33]–[35]. Although about one fourth (20–25%) NAC genes functions in at least one or the stress-response [6], [9], [36]–[37], very few candidate genes have been functionally characterized for enhancement of stress tolerance [14], [36], [38]–[48].

Correspondingly, till now foxtail millet invited little research with respect to development of genetic, genomic and functional resources although it is potentially better stress tolerant when compared to other staple cereals. The recent release of its genome sequence facilitates the prediction and systematic analysis of important genes families, including the multi-functional plant-specific NAC TFs. In this context, we conducted a genome-wide survey and identified a comprehensive and non-redundant set of 147 *NAC* genes from foxtail millet (internally annotated as *Setaria italica NAC*; *SiNAC*) and classified into eleven classes on basis of the conserved motifs and sequence phylogeny. Sequence comparison of *SiNAC* genes with themselves and with other monocots like sorghum, maize and rice facilitated the detection of presence and distribution of paralogous and orthologous *NAC* genes between the grasses. The experimental outcomes have paved a way for further comparative genomic and phylogenetic analyses of NAC TFs among members of grass family. Subsequently, quantitative real-time PCR (qRT-PCR)-based gene expression profiling displayed temporal and stress-specific expression pattern of selected candidate *SiNAC* genes. Three-dimensional structure determination and molecular simulation of a stress-responsive protein SiNAC128 was performed for understanding the basis of its molecular function. This study provides the first information about foxtail millet NAC genes, which would serve as potential candidates for dissecting NAC-mediated regulatory pathways in this significant yet neglected crop species.

## Materials and Methods

### Sequence Retrieval and Identification of NAC Domain Proteins from *Setaria italica*

Three different approaches were applied to identify putative NAC domain containing proteins from *Setaria italica*. Initially, 601 amino acid sequences encoding NAC transcription factors from four plants (*Arabidopsis thaliana*, *Oryza sativa*, *Zea mays* and *Sorghum bicolor*) were retrieved from plant transcription factor database 3.0 (plntfdb.bio.uni-potsdam.de/) [49]. These sequences were used to identify homologous peptides from foxtail millet by performing a BLASTP search at PHYTOZOME v8.0 database (www.phytozome.net/) using default parameters [2], [50]. In addition, the database was searched using the keywords ‘NAC’, ‘no apical meristem’ or ‘NAM’’. Moreover, the HMM profiles of the NAM and NAC domains in the Pfam database (http://pfam.sanger.ac.uk/) were searched against the PHYTOZOME database of *Setaria italica*. Similarity searches were also performed through TBLASTN at NCBI database against the EST sequences of *S. italica* genome to eliminate possible exclusions of any additional NAC member. All hits with expected values less than 1.0 were retrieved and redundant sequences were removed using the decrease redundancy tool (web.expasy.org/decrease_redundancy). Each non-redundant sequence was checked for the presence of the conserved NAC domain by SMART (http://smart.embl-heidelberg.de/) [51] and Pfam (http://pfam.sanger.ac.uk/) searches. Transmembrane motifs in the sequences were identified with TMHMM Server v.2.0 (http://www.cbs.dtu.dk/services/TMHMM/) using default parameters.

### Chromosomal Location, Gene Structure and Estimation of Genomic Distribution

Specific chromosomal position of the genes encoding these SiNAC proteins were determined by BLASTP search of the *Setaria italica* sequences against the PHYTOZOME database using default settings. The genes were plotted separately onto the nine foxtail millet chromosomes according to their ascending order of physical position (bp), from the short arm telomere to the long arm telomere and finally displayed using MapChart [52]. As a gene family may be expanded through tandem and segmental duplication events, we intended to identify the mechanisms involved for expansion of NAC members in foxtail millet. Segmental duplications were identified based on the method of Plant Genome Duplication Database [53]. Briefly, BLASTP search was executed against all predicted peptide sequences of *Setaria italica* and top 5 matches with E-value<1e-05 were identified as potential anchors. Collinear blocks were evaluated by MCScan and alignments with E-value<1e-10 were considered as significant matches [53], [54]. Tandem duplications were characterized as adjacent genes of same sub-family located within 10 predicted genes apart or within 30 kb of each other [54], [55]. The exon-intron organizations of the genes were determined using Gene structure display server (gsds.cbi.pku.edu.cn/) [56] through comparison of their full-length cDNA or predicted coding sequence (CDS) with their corresponding genomic sequence.

### Sequence Alignment, Phylogenetic Analysis and Identification of Conserved Motifs

The amino acid sequences were imported into MEGA5 [57] and multiple sequence alignments were performed using ClustalW with a gap open and gap extension penalties of 10 and 0.1, respectively [58]. The alignment file was then used to construct an unrooted phylogenetic tree based on the neighbor-joining method [59] and after bootstrap analysis for 1000 replicates, the tree was displayed using Interactive tree of life (iTOL; http://itol.embl.de/index.shtml) [60]. Protein sequence motifs were identified using the multiple EM for motif elicitation (MEME); (http://meme.nbcr.net/meme3/meme.html) [61]. The analysis was performed by keeping number of repetitions, any; maximum number of motifs, 20; and optimum width of the motif, ≥50. Discovered MEME motifs (≤1E-30) were searched in the InterPro database with InterProScan [62].

### Comparative Physical Mapping of SiNAC Proteins between Foxtail and other Grass Species

For deriving orthologous relationship among the chromosomes of foxtail millet and three other grass species amino acid sequences of SiNAC, that were physically mapped on the nine chromosomes of foxtail millet, were searched against peptide sequences of sorghum, maize and rice (http://gramene.org/; www.phytozome.net) using BLASTP. Hits with E-value≤1e-5 and atleast 80% identify were considered significant. The comparative orthologous relationships of *NAC* genes among foxtail millet, rice, sorghum and maize chromosomes were finally visualized using MapChart.

### Estimation of Synonymous and Non-synonymous Substitution Rates

CLUSTALW-based multiple sequence alignment was performed using the amino acid sequences of the duplicated genes as well as orthologous gene pairs between foxtail millet and rice, maize and sorghum. The aligned amino acid sequences and their corresponding original cDNA sequences were analysed using the CODEML program in PAML interface tool of PAL2NAL (http://www.bork.embl.de/pal2nal/) [63], to estimate the synonymous (Ks) and non-synonymous (Ka) substitution rates. Time (million years ago, Mya) of duplication and divergence of each SiNAC genes were calculated using a synonymous mutation rate of λ substitutions per synonymous site per year as T = Ks/2λ (λ = 6.5×10^−9^) [64], [65].

### Plant Materials and Treatments

Seeds of foxtail millet cultivar Prasad obtained from National Bureau of Plant Genetic Resources (NBPGR), Hyderabad, India were grown in a plant growth chamber (PGC-6L; Percival Scientific Inc., USA) at 28±1°C day/23±1°C night with 70±5% relative humidity and photoperiod of 14 h. For stress treatments, 21-day-old seedlings were exposed to 250 mM NaCl (salinity), 20% PEG 6000 (dehydration), 100 µM abscisic acid (ABA), 100 µM salicylic acid (SA), 100 µM methyl jasmonate (MJ) or 100 µM ethephon (Et) for 1 h (early) and 24 h (late) based on earlier studies [10], [22], [66]. Unstressed plants were maintained as controls. After the treatments, seedlings were immediately frozen in liquid nitrogen and stored at −80°C until RNA isolation as described elsewhere [66]. For obtaining precise and reproducible results, each of these above experiments was repeated twice.

### RNA Extraction and qRT-PCR Analysis

Total RNA was extracted according to Longeman et al. [67]. DNA contamination was removed from the RNA samples using RNase-free DNase I (50 U/µl; Fermentas, USA). The quality and purity of the preparations were determined at OD_260_:OD_280_ nm absorption ratio (1.8–2.0) and the integrity of the preparations was ascertained by electrophoresis in a 1.2% agarose gel containing formaldehyde. One µg total RNA was reverse transcribed to first strand cDNA using random primers by Protoscript M-MuLV RT (New England Biolabs, USA) following manufacturer’s instructions. The qRT-PCR primers were designed from non-conserved regions of the genes using Primer Express 3.0 software (PE Applied Biosystems, USA) with default parameters (Table S1). qRT-PCR was performed in three technical replicates for each biological duplicate using one step real-time PCR system (Applied Biosytems, USA). The PCR mixtures and reactions were used as described elsewhere [68]. Melting curve analysis (60 to 95°C after 40 cycles) and agarose gel electrophoresis were performed to check the amplification specificity for absence of multiple amplicons or primer dimers [69]. A constitutive 18S-rRNA gene-based primer was used as endogenous control. The amount of transcript accumulated for *SiNAC* genes normalized to the internal control 18S-rRNA were analyzed using 2^–ΔΔ^Ct method. cDNA synthesis and qRT-PCR analysis were performed according to Jayaraman et al. [70]. The PCR efficiency which is dependent on the assay, performance of the master mix and quality of sample, was calculated as: Efficiency = 10 ^(−1/slope)^–1 by the software itself (Applied Biosystems).

### Structure Modeling, Molecular Simulation and Docking Analysis

All the SiNAC proteins were searched against the Protein Data Bank (PDB) [71] by BLASTP (with the default parameters) to identify the best template having similar sequence and known three-dimensional structures. Secondary structure prediction of SiNAC proteins was performed using SOPMA secondary structure prediction method (http://npsa-pbil.ibcp.fr/cgi-bin/npsa_automat.pl?page=/NPSA/npsa_sopma.html) [72]. Structures which had very high sequence identity with the query sequence (> 95%) were used as template for three dimensional homology modeling. We found SiNAC128, to be highly similar to rice stress-responsive NAC1 (SNAC1; PDB ID: 3ULX). The protein structure modeling program, MODELLER v. 9.10 [73] was applied for automated homology model building of SiNAC128 as described by Puranik et al. [22]. The model structure was validated using PROSA [74] and subjected to further refinement by loop modelling using the ModLoop server (http://modbase.compbio.ucsf.edu/modloop/) [75]. Molecular dynamics (MD) simulation and energy minimization of the refined model was executed by Gromacs v. 4.5.5 (GROningen MAchine for Chemical Simulations) [76]. The stability of the structure was observed by plotting RMSD values in GNUPLOT software (http://www.gnuplot.info/index.html). After the structure was stabilized by simulation, the DNA oligomer of 4 nucleotides was retrieved from PDB (PDB id: 1ANA; sequence IC-C-G-G). Docking of 1ANA with the protein model was executed by under default parameters at HexServer (http://hexserver.loria.fr/) [77]. The resultant best docking solution was visualized by applying PYMOL (http://www.pymol.org/) and DS visualizer (http://accelrys.com/products/discovery-studio/visualization.html). The docking complex was analyzed by observing the polar and hydrophobic amino acids around 5 Å of the 1ANA.

## Results and Discussion

### Genome-wide *in silico* Survey and Identification of Novel *SiNAC* Members from Foxtail Millet

The keyword, HMM profile and BLAST searches predicted that the *Setaria italica* genome encodes about 147 NAC proteins. Due to no proper annotation, the existing identifies for these genes were highly disordered. We, therefore, internally assigned them a consecutive numbering based on the order of their chromosomal locations: SiNAC001-SiNAC147 for the convenience of research community (Table S2). All the SiNAC proteins varied greatly in their lengths, in positions of the conserved NAC domains as well as in their subcellular localization with most being nuclear localized. Five proteins (SiNAC016, SiNAC049, SiNAC054, SiNAC090 and SiNAC092) were found to be splice variants of primary transcripts encoding NAC proteins (SiNAC015, SiNAC050, SiNAC055 and SiNAC091), and hence, only the latter were selected for phylogenetic and comparative analyses.

**Figure 1:**
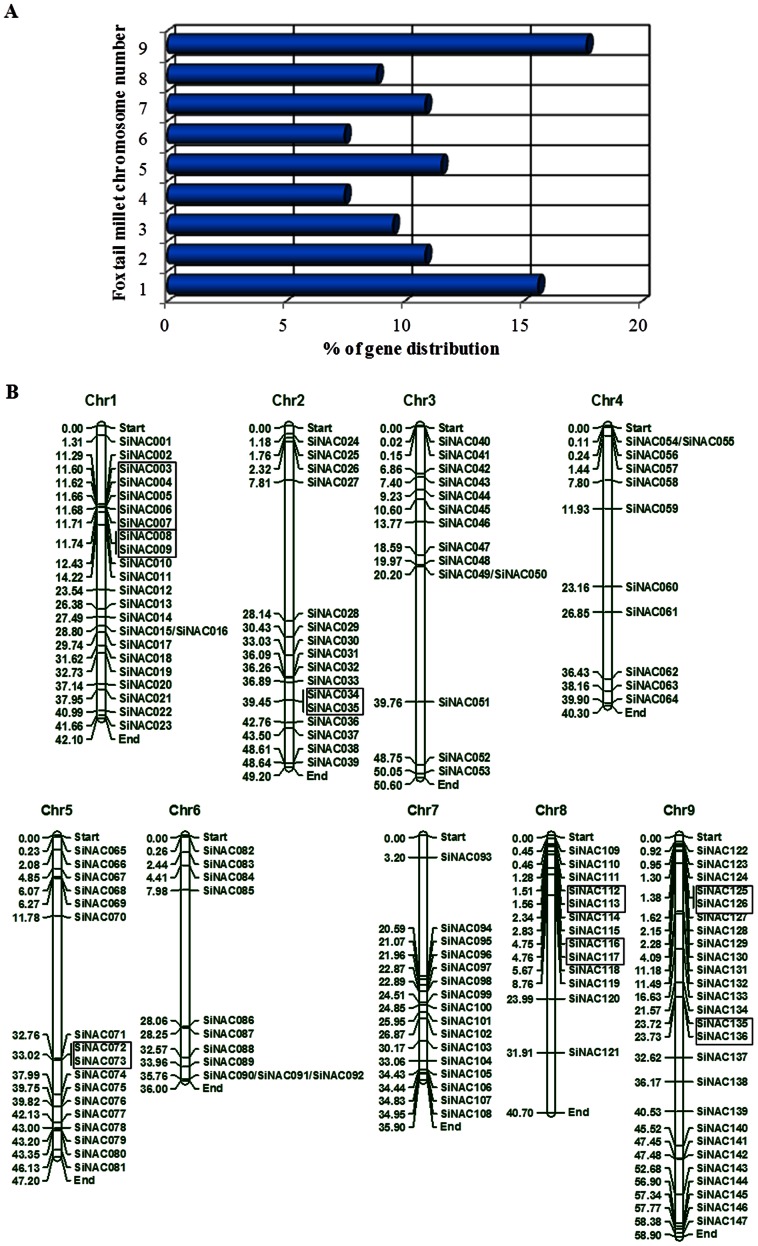
Distribution of 147 *SiNAC* genes onto nine foxtail millet chromosomes. (A) Percentage of *SiNAC* genes on each the foxtail millet chromosome to show their distribution abundance. (B) Graphical (scaled) representation of physical locations for each *SiNAC* gene on foxtail millet chromosomes (numbered 1–9). Tandem duplicated genes on a particular chromosome are depicted by specific color lines. Chromosomal distances are given in Mb.

Typically, the NAC proteins have N-terminal conserved domain (for DNA- or- binding) and a C-terminal variable domain. Recently, it was described that these, certain atypical NACs also exist (For details see [5]: Figure 1A). Out of the 147 proteins, 110 had the general structure of NAC proteins and were classified into structure group ‘i’ (Table S2). Eight proteins (SiNAC023, SiNAC031, SiNAC055, SiNAC067, SiNAC084, SiNAC090, SiNAC091 and SiNAC092, group: ii) were predicted to comprise a single trans-membrane (TM) region at their C-terminal ends. Structure group-iii included 6 proteins containing only the NAC domains while those of group-iv (2 proteins) have two tandemly repeated NAC domains. The typical orientation of NAC proteins was completely reversed in SiNAC129 and an AT-hook domain preceded the C-terminal NAC domain. A plant defense-responsive AAA-ATPase domain/ NB-ARC domain was identified in SiNAC115 indicating a putative involvement of this protein in biotic stress response.

### Chromosomal Distribution and Structure of *SiNAC*

The genome of foxtail millet comprises of nine chromosomes varying in their length, shortest being chromosome 7 (35.9 Mb) and longest is the chromosome 9 (58.9 Mb). *In silico* mapping of *SiNAC*s on chromosomes indicated an uneven distribution of the genes on all the 9 chromosomes of foxtail millet (Figure 1A,B). Among all, chromosome 9 contains the highest number of *SiNACs* [26 (∼18%)], while minimum genes were distributed on chromosomes 4 and 6 (11 each; ∼7%) (Figure 1A). The exact position (in bp) of each *SiNAC* on foxtail millet chromosome is given in Table S2. Pattern of their distribution on individual chromosomes also revealed certain physical regions with a relatively higher accumulation of *SiNAC* gene clusters. For example, *SiNAC* genes located on chromosomes 7 and 8 appear to be congregate at the lower end and upper end of the arms, respectively (Figure 1B). It was recently reported that the foxtail millet genome underwent whole-genome duplication (WGD) similar to other grasses ∼70, Myr ago [1]. Hence, the presence of such large number of *SiNAC* genes in foxtail millet indicates the amplification of this gene family during the course of evolution. In all, 19 (∼13%) *SiNAC* genes were found to be tandem repeats (Figure 1B). This included eight clusters of tandemly repeated *SiNAC*s (2 to 5 genes) including two clusters on chromosome 1, 8 and 9 and one each on chromosomes 2 and 5. Absence of any segmentally duplicated SiNAC gene suggests that the SiNAC gene family has primarily expanded through tandem repetitions rather than by segmental duplication.

Investigation of *SiNAC* gene structures revealed highly diverse distribution of intronic regions (from 0 to 12 in numbers) amid the exonic sequences signifying considerable evolutionary changes in the foxtail millet genome. The shortest *SiNAC* gene was merely 537 bp (*SiNAC139*) whereas the longest one was identified as *SiNAC115* with ∼8.7 kb genomic sequence. Further, 26 genes (∼18%) possessed no introns, and 11 (∼42%) of these intron-less genes were tandem repeats (Table S2, Figure S1). This infers that the evolution of these genes might have proceeded quickly through some gene duplications or by integration into genomic region after reverse transcription [78]–[80].

### Phylogenetic Classification of SiNACs and Identification of Motif Conservation

Sequence analysis of the deduced SiNAC proteins showed that most of the SiNAC TFs shared a highly conserved typical NAC domain containing five consensus subdomains and a highly variable C-terminal transcriptional regulation domain [37], [81]. SiNAC44 being a partial peptide sequence was excluded from alignment and phylogenetic tree construction being a partial peptide sequence. Apart from these, certain NAC proteins did not share much similarity with their usually conserved structures as described earlier. Such variation may be a result of gene duplication and/or recombination that leads to domain rearrangements.

**Figure 2:**
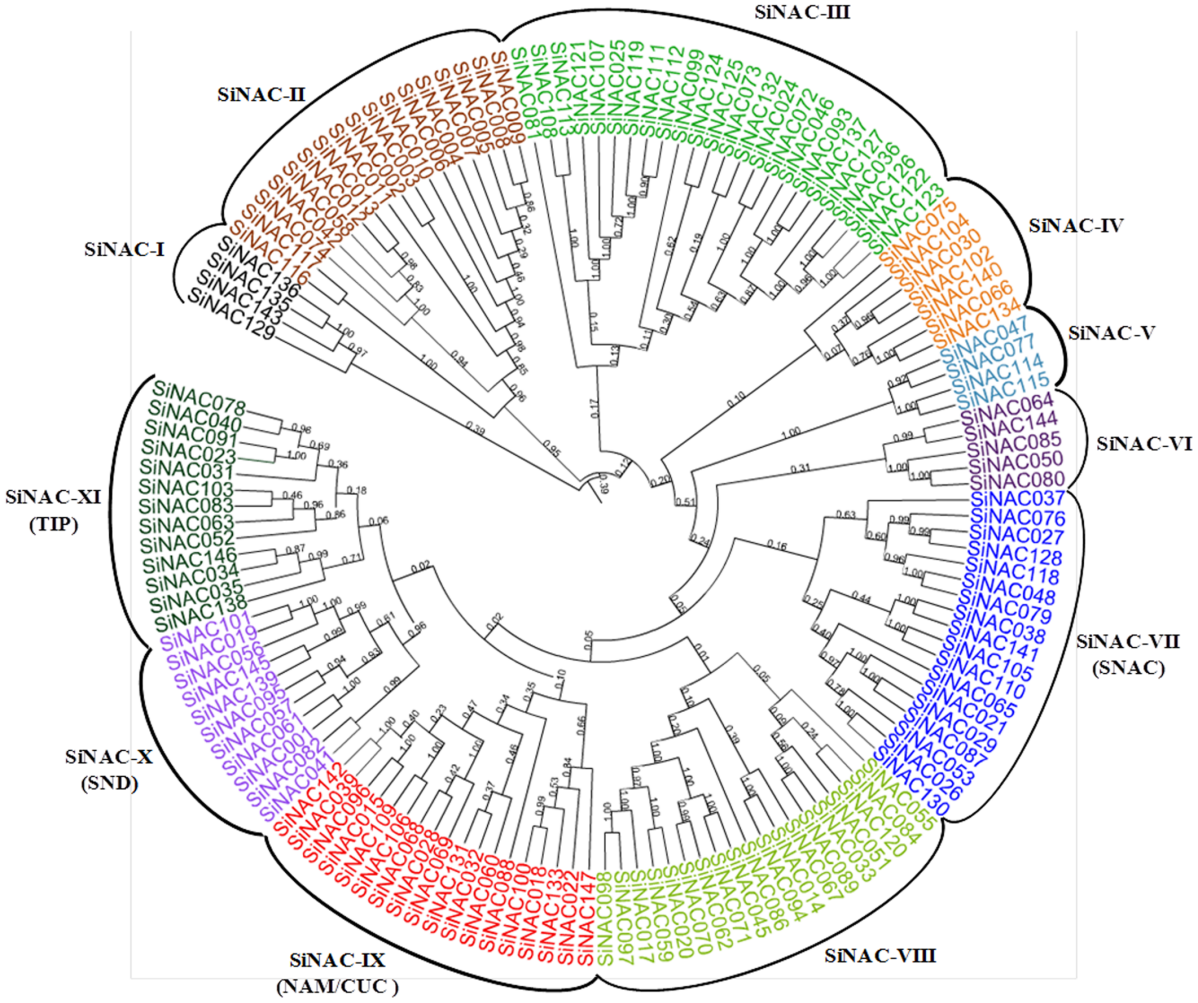
Phylogenetic relationships of foxtail millet NAC proteins. The sequences were aligned by CLUSTALW at MEGA5 and the unrooted phylogenetic tree was deduced by neighbor-joining method. The proteins were classified into eleven distinct sub-families (SiNAC-I to SiNAC-XI). Each sub-family was assigned a different color according to well-known members in other species.

To understand the evolutionary significance of domain architecture in SiNAC proteins, we performed a comprehensive phylogenic analysis. This enabled the classification of SiNAC family into eleven major sub-families, denoted as SiNAC-I to SiNAC-XI, each in turn being composed of several members (Figure 2). Close association of SiNAC families with their counter-parts in other plants, expressions and/or functions for most of which have reported, may be an implication of sequence conservation and evidence to their similar biological *in planta* roles. Being a rational systematic approach, such phylogeny-based function prediction has near-perfectly been applied for prediction of stress-responsive NAC proteins in other species like rice, *Arabidopsis* and soybean [9], [10], [37], [82]. Thus, members of the subfamilies SiNAC-IX, SiNAC-X, SiNAC-XI and SiNAC-VII are probably involved in similar regulatory roles as those of their orthologous groups namely NAM/CUC, SND, TIP and SNAC (stress-responsive NACs), respectively. Separation of 4 proteins classified under sub-group SiNAC-I from the rest of the sub-families suggests their origin by an early divergence event. Statistical significance of the phylogenetic analysis was confirmed through bootstrap analysis of 1000 replicates. A good number of the internal branches had high bootstrap values reflecting derivation of statistically reliable pairs of possible homologous proteins sharing similar functions from a common ancestor.

Interestingly, the eight predicted TM region-containing SiNACs belonged either to the sub-family SiNAC-VIII or to SiNAC-XI (TIP). Previous genome-wide analysis of this family has predicted at least 18 membrane-associated NAC transcription factors (MTFs) in Arabidopsis, 5 in rice and 11 in soybean [10], [83]. Their phylogenetic comparison with membrane-associated NAC transcription factors (MTFs) of Arabidopsis, rice and soybean NAC proteins showed *SiNAC* MTFs to be closely associated with rice MTFs (Figure S2). NAC MTFs have been established as transcription regulators which specifically get activated via post-translational modifications during environmental stresses. Therefore, SiNAC MTFs may follow a similar path of nuclear localization and downstream stress-responsive gene expression only after their membrane anchors have been trimmed by proteases following adverse environmental cues.

Fascinatingly, all those genes identified as tandem duplications got assigned under similar sub-groups with high bootstrap values, indicating their common but delayed origin from one single ancestor. For example, SiNAC003 to SiNAC009, which are proximally arranged on chromosome 1 were found to be clustered together in subgroup SiNAC-II. Such arrangements have been also reported by other research groups in different plant genomes [37], [84], [85]. Additionally, reliability of the phylogeny was further evidenced by parameters like motif compositions of individual sub-families and MEME analysis identified 15 motifs (Table 1, Figure S3), of which 7 were located in the conserved NAC domain region. Such motif sequence conservation or variation between the proteins specifies the functional equivalence or diversification, respectively, with respect to the various aspects of biological functions [5]. As reported in some earlier studies, members of a particular sub-family showed a tendency to have comparable motif composition (with slight variations) [8], [37], [85], and certain motifs were found to be deleted or duplicated within particular clades. Noticeably, motifs 1 and 6 were absent from almost all the members of sub-family SiNAC-II and instead included the motif 10 and 12. Motif deletion or duplication within a conserved domain of a protein may be crucial for disposing the undesirable regions and developing only the regions which are necessarily needed to develop a particular phenotype.

**Table 1:** Conserved motifs identified in foxtail millet NAC family proteins by MEME software.

Motif No.	Sites	E-value	Amino acid sequence composition of motif	Width (aa)	NACSubdomain
Motif 1	96	3.2e-1139	W[YF]FF[SC]P[RK]DRKYP[TN]GSR[TP]NRAT	21	C
Motif 2	122	4.5e-1131	LPPGFRFHPTDEEL[VI]x[HYF]	17	A
Motif 3	139	2.7e-1039	P[KR]Gx[KR]T[GD]W[VI]MHEYRL	15	D
Motif 4	73	8.0e-747	IA[ED]VD[LI][YN][KR]C[ED]PW[DE]LP[ED]KAKIG	21	B
Motif 5	93	4.6e-766	[VI]xxGGRL[VI]GM[KR]KTLVFYRGRA	21	D
Motif 6	107	6.5e-554	xx[DE][DE]WV[LV]C[RK][VI][FY]K[KS]PR	15	E
Motif 7	78	1.7e-519	[SA]G[YF]WKATGKD[KR]	11	C
Motif 8	80	8.1e-244	YL[RK]RK[VA]AGx[PR][LI]PLD[VI]	15	–
Motif 9	11	2.2e-243	[RV][PH][VF]V[HN]H[AV]DV[YC][GS][CA][EA]P[AE]DL[AV][RA][QD][FL][CE]P[LA]P[RG]T[GS][HV][RW][FY]F[FY][TC][HV][CK]K[LY][QK][QS][PT][HQ][RG][AR][GP][KG][AG][SH]R[AQ]	50	–
Motif 10	23	1.5e-227	[FC][PS][FI][LDV][KQR][IG][CI][HS][AVI][MT][FT][TH][GQ][HQ][GQ][KM][KVW][RM][KM][RP][MV]PDD[EK][SN]DCQ	29	–
Motif 11	6	2.0e-226	FFVHTNNEVARQDRYCPGDGTWVSQRQESGSSCICGETIKWRRTNLNLQM	50	–
Motif 12	6	7.3e-176	NSSSATCA[YN]GSTMTTADQDSGAAHAYAGEESAQDTDEETLEWFRLDGKDL	50	–
Motif 13	21	1.7e-174	I[LD]DD[DS][PA][LA][SN][AT][LP]PW[EN]LL[EK]R[HN]G[LR][KV]	21	–
Motif 14	6	6.6e-150	AAPKRPAPQSAEPPCPKRMRGAVAPTPPVVQPAGYCTASFAPPLPY	46	–
Motif 15	8	4.4e-179	[VL][VE][RK][AC][CM][HD][MD][PA]V[EPQ][AT][PA][AE][RG][HS][CT][QV][PS][PE][QD][PE][SM][VD]Q[RTK]KQST[RD]DPFEAAEL[RG]DEAE[EK]E[RS]VAAP	50	–

### Orthologous Relationships of NAC TF Genes between Foxtail Millet and other Grass Species

For comparative mapping to derive orthologous relationships of SiNACs, the physically mapped SiNAC genes were compared with those in chromosomes of other related grass genomes namely, sorghum, maize and rice (Figure S4A–C). Although rice, sorghum and maize genome encodes more than 100 NAC proteins, the specific orthologous relationships of NACs could be derived only for ∼31% proteins. Highest orthology of genes annotated on the foxtail millet chromosomes was exhibited with sorghum (76.5%) and maize (72%). The extensive gene level synteny shared among foxtail millet, sorghum and maize supports their close evolutionary relationships [1], [2]. Interestingly, they did not reveal biasness for any particular chromosome of maize and were instead randomly distributed. A plausible reason for this might be the higher synteny and collinearity of genes annotated on the foxtail millet chromosomes with rice (69.5%) and sorghum (65.2%) chromosomes than to maize chromosomes (32.1%) at genome-wide level [1].

Noticeably, the SiNACs on foxtail millet chromosome 6 showed 100% orthology and synteny with sorghum chromosome 7 and rice chromosome 8 with certain differences in chromosomal arrangements, predominantly involving intra-chromosomal inversions (Table 2, Figure S4A–C). For example, orthologs of SiNAC139 and SiNAC145 had inverse orientation in chromosome 1 of sorghum (Sb01g030760 and Sb01g048730) but are arranged forward orientation in maize (chromosome 1; GRMZM2G025642 and GRMZM2G025642) (Figure S4B and C). SiNAC031, SiNAC033, SiNAC035 on foxtail millet chromosome 2 appear to have exactly the same orientation as their maize homologs (Figure S4B). These results indicate that the tendency of homologous genes to occupy similar relative organization may have co-developed from an evolutionarily homologous genomic region as compared to those which have re-located to different site on chromosome [86], [87]. The results also indicated that the chromosomal rearrangements like duplication, inversion and deletion were predominant in shaping the distribution and organization of NAC genes in foxtail millet, rice and sorghum genomes. This comparative mapping provides a useful preface for understanding the evolutionary process of NACs among grasses involving the foxtail millet genome. The knowledge thus generated would also be useful in isolating orthologous NAC genes of agronomic importance from foxtail millet using the map-based genomic information of other related small and diploid grass members. Otherwise, based on this comparative genome map information, some candidate SiNAC genes (for example, SiNAC128) can be rapidly selected from the genome of this naturally stress-adapted crop and utilized for genetic enhancement of other related grass family members for target traits like stress tolerance.

**Table 2:** A summary of comparative mapping of foxtail millet SiNAC genes on sorghum, maize and rice.

Setaria italica	Oryza sativa	Sorghum bicolor	Zea mays
Chr1 (23)	–	Chr4 (100%)	Chr5 (66.67%)
Chr2 (16)	Chr9 (100%)	Chr2 (100%)	Chr7 (75%)
Chr3 (14)	–	Chr5/6/9 (33.33%)	Chr6/8 (50%)
Chr4 (11)	Chr6 (100%)	Chr10 (100%)	Chr5/6/9 (16.67%/50%/16.67%)
Chr5 (17)	Chr1 (75%)	Chr3 (100%)	Chr3/4/8 (25%/25%/ 50%)
Chr6 (11)	Chr8 (100%)	Chr7 (100%)	Chr4/6 (66.67%/33.33%)
Chr7 (16)	–	Chr6 (100%)	Chr2/4/6 (33.33%)
Chr8 (13)	Chr1 (100%)	Chr9 (100%)	Chr6 (100%)
Chr9 (26)	Chr3 (100%)	Chr1 (87.5%)	Chr1/5/9 (50%/12.5/37.5%)

### Duplication and Divergence Rate of the SiNAC Genes

**Figure 3:**
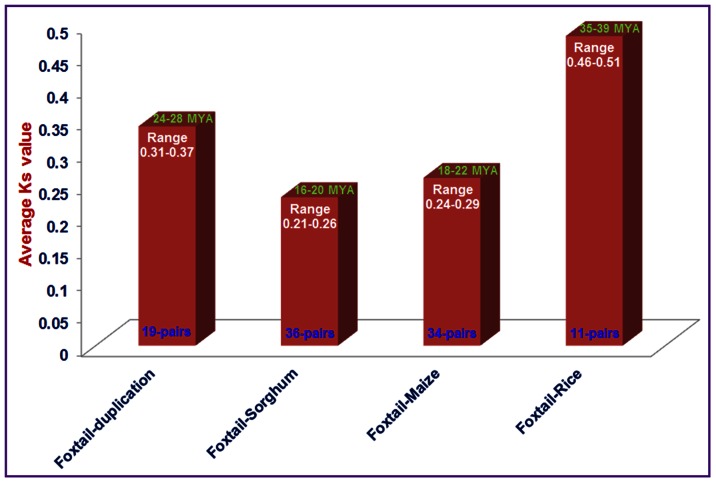
Time of duplication and divergence (MYA) based on synonymous substitution rate (Ks) estimated using 19 duplicated SiNAC gene pairs of foxtail millet and orthologous SiNAC gene pairs between foxtail millet and rice (11) or maize (34) or sorghum (36).

Multiple members of a gene family could possiblly evolve due to the flexibility provided by events of whole genome duplications. Gene duplication, either segmental or tandem, have been found in several plant TF families such as MYB, F-box as well as in NAC [9], [69], [88], [89]. We thus explored association of Darwinian positive selection in divergence and duplication of NAC genes. For this, the ratios of non-synonymous (Ka) versus synonymous (Ks) substitution rate (Ka/Ks) were calculated for 19 tandemly duplicated genes as well as between orthogous gene pairs of SiNACs with those of rice (11-pairs), maize (34) and sorghum (36). The ratios of Ka/Ks for tandem duplication varied from 0.03 to 0.13 with an average of 0.06 (Table 3). This analysis shows that the SiNAC gene family had strong purifying selection pressure as Ka/Ks ratios of the duplicated genes were <1 and the duplication event may be estimated to have occurred around 24–28 Mya (Figure 3, Table 3). Among the orthologous gene-pairs of SiNAC with those of other grass species, the average Ka/Ks value was highest between rice and foxtail millet (0.56) and least for sorghum-foxtail millet gene-pairs (0.22; Table S3). The relatively higher rate of synonymous substitution between rice and foxtail millet NAC genes may point towards their earlier divergence around 35–39 Mya from foxtail millet as compared to sorghum and maize NAC genes (Figure 3). Conversely, the NAC gene-pairs between sorghum and foxtail millet (average Ka/Ks = 0.22) seem to have largely encountered intense purifying selection as compared to foxtail millet-maize (Ka/Ks = 0.31) and foxtail millet-rice (Ka/Ks = 0.56) NAC genes, which agreed well with their recent time of divergence around 16–20 Mya. The estimation of duplication time (average of 26.2 Mya) of foxtail millet NAC genes in between the divergence time of foxtail millet-rice (36.9 Mya) and foxtail millet-maize (20 Mya) and –sorghum (17.7 Mya) orthologous NAC gene-pairs are comparable to evolutionary studies involving the protein-coding genes annotated from the recently released draft genome sequence of foxtail millet [1].

**Table 3:** The Ka/Ks ratios and estimated divergence time for tandemly duplicated SiNAC proteins.

Name	Ks	Ka	Ka/Ks	Duplication time(million years ago)
SiNAC003	0.32	0.04	0.13	24.6
SiNAC004	0.33	0.02	0.06	25.4
SiNAC005	0.34	0.03	0.09	26.2
SiNAC006	0.35	0.02	0.06	26.9
SiNAC007	0.35	0.03	0.09	26.9
SiNAC008	0.36	0.01	0.03	27.7
SiNAC009	0.34	0.04	0.12	26.2
SiNAC034	0.31	0.02	0.06	23.8
SiNAC035	0.33	0.04	0.12	25.4
SiNAC072	0.32	0.03	0.09	24.6
SiNAC073	0.34	0.02	0.06	26.2
SiNAC112	0.36	0.03	0.08	27.7
SiNAC113	0.35	0.04	0.11	26.9
SiNAC116	0.34	0.04	0.12	26.2
SiNAC117	0.36	0.01	0.03	27.7
SiNAC125	0.34	0.04	0.12	26.2
SiNAC126	0.36	0.03	0.08	27.7
SiNAC135	0.36	0.04	0.11	27.7
SiNAC136	0.35	0.04	0.11	26.9
Average	0.34	0.03	0.09	26.2

### SiNAC Expression Profiles of during Abiotic Stresses and Phytohormone Treatments

**Figure 4:**
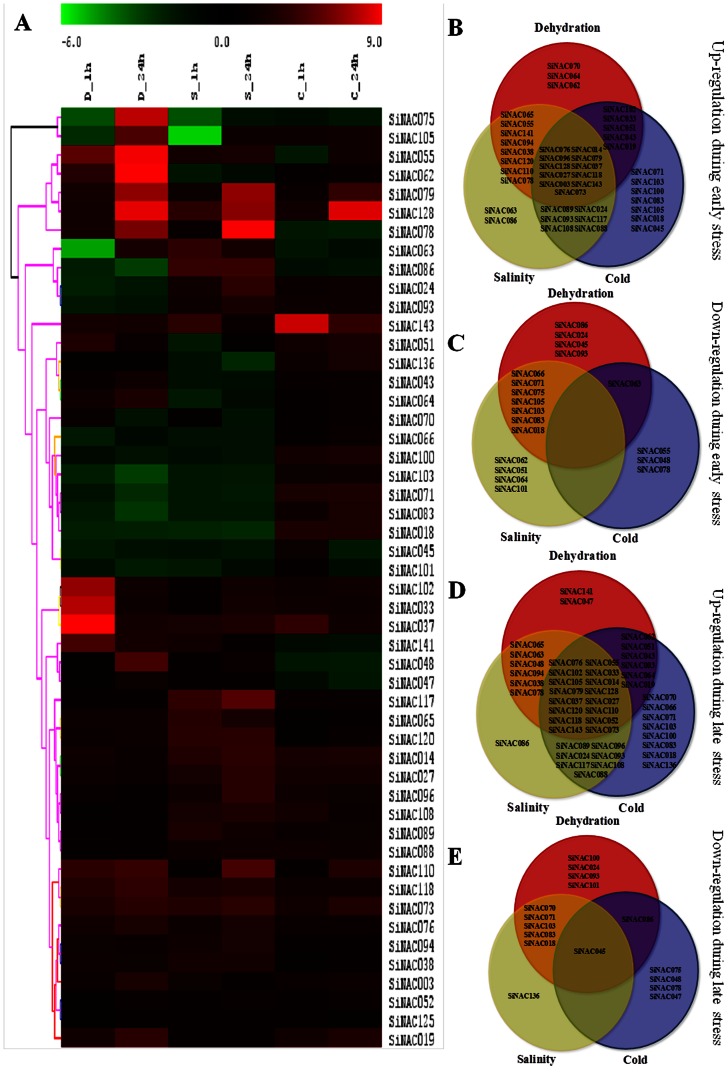
Expression profile of 50 foxtail millet *NAC* genes in response to various abiotic stresses. (A) Hierarchical clustering of differential gene expression in response to dehydration (D), salinity (S) and cold (C) stress across two time points (1 h and 24 h). The heat-map has been generated based on the fold-change values in the treated sample when compared with its unstressed control sample. The color scale for fold-change values is shown at the top. Venn diagram showing stress-specific distribution of *SiNAC* genes into four categories as (B) early up-regulated, (C) early down-regulated (D) late up-regulated, and (E) late down-regulated. The common subset of genes regulated by two or more stresses is marked by the overlapping circle.

**Figure 5:**
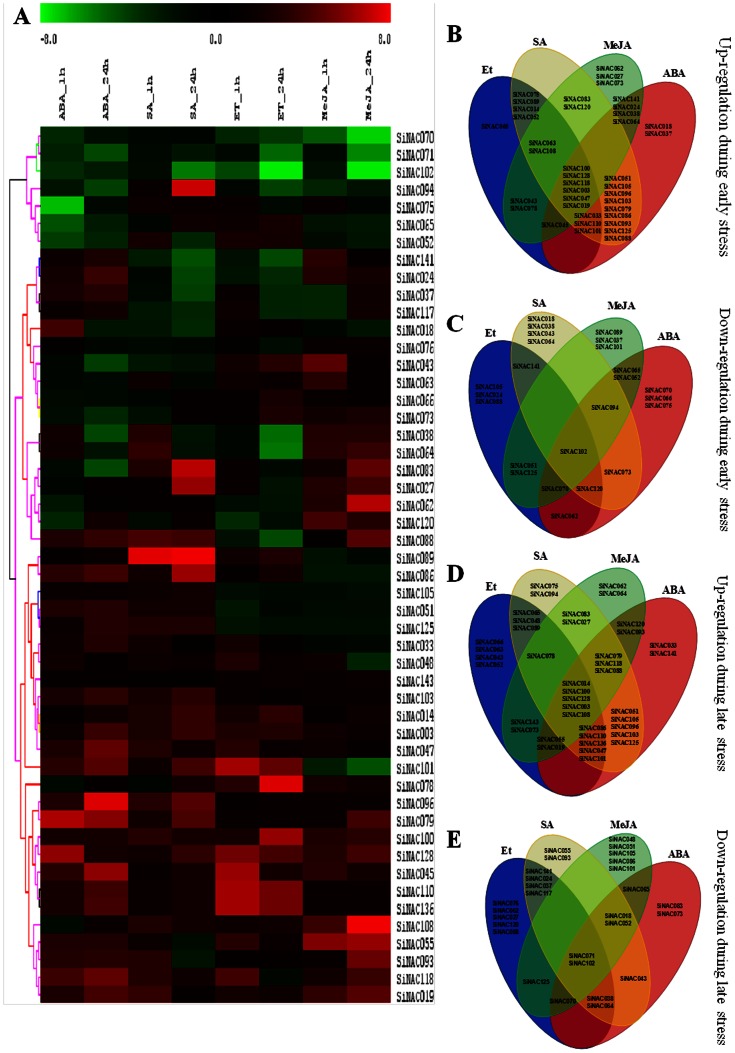
Expression profile of 50 foxtail millet *NAC* genes in response to various hormones. (A) Hierarchical clustering of differential gene expression in response to salicylic acid (SA), Ethephone (ET), Absissic acid (ABA) and methyl jasmonate (MeJA) stress across two time points (1 h and 24 h). The heat-map has been generated based on the fold-change values in the treated sample when compared with its unstressed control sample. The color scale for fold-change values is shown at the top. Venn diagram showing stress-specific distribution of *SiNAC* genes into four categories as (B) early up-regulated, (C) early down-regulated (D) late up-regulated, and (E) late down-regulated. The common subset of genes regulated by two or more stresses is marked by the overlapping circle.

Gene expression patterns can provide crucial clues for determining the gene function. In order to decipher the role of NAC genes in foxtail millet during diverse environmental conditions, 50 candidate genes, widely representing all the sub-families, were subjected to quantitative expression analysis in response to dehydration, salinity, cold, ABA, SA, MeJA and Et during early and late durations of treatments (Figure 4 and 5, Table S4). We included a previously reported stress-responsive gene, *SiNAC078*, in the qRT-PCR analysis to serve as a positive reference for verification of the experiments [22]. The heat map representation for expression in response to abiotic stresses like dehydration, salinity and cold is shown in Figure 4A. Overall, qRT-PCR analysis demonstrated that all the genes displayed variations in their expression behavior in response to one or more stresses in course of the experimentations (Figure 4B–E). Among all the three treatments, cold stress induced relatively more dramatic changes in transcript abundance of *SiNAC* than dehydration or salinity. Some genes were identified to be differentially expressed in response to a specific stress treatment at only one time. For example, *SiNAC141* was early induced by both dehydration and salinity while during later time-point only dehydration was able to induce its expression. On the basis of their expression during both early and late durations, many genes were up-regulated by all stresses at either time points (Figure 4B and D), but none was down-regulated by every stress except *SiNAC045* (Figure 4C and E). The results also revealed that some genes are co-regulated by two stresses, for example, transcripts of *SiNAC024* and *SiNAC093* accumulated only during salinity and cold treatments. Notably, *SiNAC045* could be a late stress-responsive gene as it was exclusively up-regulated by dehydration, salinity as well as cold only at 24 h post-stress. Previously, several whole-genome expression profiling studies in *Arabidopsis* and rice have found NAC genes to be induced by at least one type of abiotic stress like salinity, drought, cold or ABA [6], [36], [37]. Expression analysis using 22 K and 44 K microarray revealed the induction of more than 45 NAC genes in abiotic and 26 against biotic stresses in rice [9]. Recently, global NAC gene expression analysis in *Arabidopsis* has shown that most of the NAC genes are responsive to salt and extreme temperatures [17], [90]. The variability in gene expression patterns implies that *SiNACs* may regulate a complex web of pathways to perform different physiological functions for acclimatizing towards multiple challenges.

Plant hormones play a crucial role in the regulation of different plant processes, such as signalling and expression during abiotic and biotic stresses. We thus attempted to evaluate the expression pattern of these 50 genes during various hormone treatments. A hierarchical clustering evidenced overlapping and specific gene expression patterns in response to phytohormones (Figure 5A). Several genes were exclusively induced (like *SiNAC003*, *SiNAC108*, *SiNAC100*, *SiNAC128*) or repressed (*SiNAC071* and *SiNAC 102*) in all the treatments (Figure 5B–E). These genes may be a part of a general hormonal response rather than being treatment-specific. For example, genes such as *SiNAC051* and *SiNAC105* showed up-regulation during all the durations of ABA and SA treatments while others like *SiNAC066*, *SiNAC063*, *SiNAC128* and *SiNAC043* were differentially induced by MeJA and Et at both the time points. Phytohormones are involved in influencing signaling response by acting in conjunction with or opposition to each other for maintaining the cellular homeostasis [91]. The NAC TFs form a complex but interesting group as important arbitrators of this process [5]. The highly differential expression profiles of *SiNAC* genes observed in this study underscore the daunting task of comprehending the global milieu associated with a stress response. However, an important outcome was comparison of their expressions patterns during multiple environmental stimuli at beginning and late durations of stress for accurate identification of prospective candidate genes. Together, above results indicate that some members of the *SiNAC* gene family show stimulus-specific and time-dependent responses and may widen the knowledge on molecular mechanism behind the action of NAC TFs in plant stress acclimatization.

### Comparative Modeling, Molecular Simulation and Docking Analysis of a Stress-responsive Protein SiNAC128

To build the homology model, BLASTP search against the protein databank (PDB) with known structures for NAC TFs was performed for each of 147 amino acids and 11 sequences with >60% identity were identified from different sub-families. Comparison of their secondary structures revealed that the sequences were quite variable (Figure S5) and this variability may be eventually responsible for imparting distinct functionality to the NAC proteins from different sub-families.

**Figure 6:**
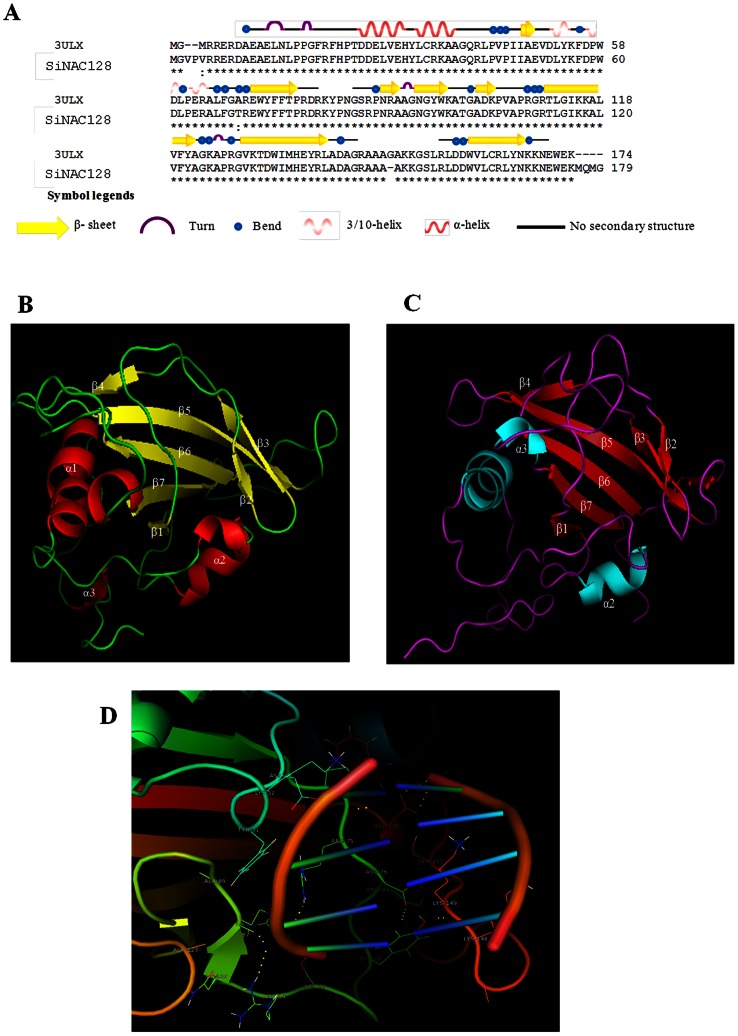
Predicated structure of SiNAC128. (A) Amino acid sequence alignment of SiNAC128 with the template 3ULX which encodes a rice stress-responsive protein 1(SNAC1). Symbol legends show the secondary structural elements of the target sequence. (B) Three dimensional structure of SiNAC128 after loop modeling showing α-helices (red) and β-sheets (yellow). (C) Ribbon diagram representation of the protein after molecular simulation. The α-helices and β-sheets are shown in blue and pink, respectively. (D) Molecular docking showing binding of the modelled protein to DNA (PDB id: 1ANA).

Among all the proteins, SiNAC128 showed 95% sequence identity (in the conserved NAC domain) with a stress-responsive SNAC1 protein from rice (PDB code 3ULX), and both the proteins shared highly analogous secondary structures (Figure 6A). Homology modeling and evaluation revealed that SiNAC128 NAC domain was fairly comparable similar to the template (SNAC1), with a semi-β-barrel core formed by seven twisted anti-parallel β-sheets, three α-helices on one side while the other side remained open (Figure S6A). PROSA validation of the generated model showed a z-score of –4.28 which follows the range of other experimentally determined protein with similar residues as SiNAC128 [74]. Low root mean square deviation (RMSD) value (0.278) showed that the template and target were quite similar. The structure quality was also assessed by Ramachandran plot which showed that 91.7% residues were energetically most favoured, 5.9% were in the allowed region and 2.4% are in outliner region. This is in accordance with the values observed for *A. thaliana* ANAC (abscisic acid-responsive NAC, PDB code 1UT7) [13]. The structure remained unaffected by certain sequence variations as most of them occurred at places where no either secondary structure had been assigned or at the loop regions between β6 and β7. Such significant conservation between the two proteins may reflect their similar biological functioning as both may share a common stress-regulatory pathway. SNAC1 crystallizes as dimer in solution, modulated by residues of the sub-domain A including Leu14 to Thr23 (N-terminal loop region), Glu26 and Tyr31 (both in α1 helix) [92]. On the basis of intact nature of these interface regions in the SiNAC128 protein, it is very likely to maintain the dimer character as its functional unit.

Model refinement was carried out using modloop server and residues 1–8, 76–84 and 141–154 corresponding to the loop regions were refined (Figure 6B). The built model was subjected to energy minimization followed by molecular dynamics (MD) simulation by Gromacs 4.5.5. The model was first minimized by steepest descent integrator method for 10000 steps followed the MD simulation for 2 ns simulation time and superimposed with pre-simulation structure (Figure 6C). The model maintained high structural conservation within the range of 1600–1900 pico seconds as observed by plotting RMSD values as a function of the simulation time in GNUPLOT software (Figure S6B). This result shows that the predicted quaternary structure of SiNAC128 NAC domain could adopt a biologically relevant stable conformation (Figure S6C and D). Property to bind DNA sequence of its target genes resides with this highly conserved domain of SiNAC128 transcriptional regulator. Docking the stable protein model against a DNA helix of 4 nucleotide base pairs (PDB id: 1ANA) was carried out in Hex Server under the default parameters. It was observed that the residues within the two of the loop regions, specifically ARG-75, ASP-76, ARG-77, TYR-79, SER-83, ARG-84, ASN-86, ARG-87, ALA-89 and LYS-148, LYS-149, SER-151, ASP-156 and TRP-157 could be responsible for interacting with 1ANA (Figure 6D). This is in accordance with previous reports where the central β-sheet of the SNAC1 and ANAC NAC domain, particularly the amino acids Lys79, Arg85, Arg88, Lys123 and Lys126 shared the responsibility of DNA binding [13], [92]. Although most of the residues were located in the loop regions which may vary among even closely homologous structures due their high flexibility [93], structural and functional resemblance between SiNAC128 and SNAC1 is conceivable given conservation of the crucial residues particularly Arg84 (Arg85 in SNAC1) and Arg87 (Arg88 in SNAC1) which have been reported to share the responsibility of DNA binding.

### Conclusions

The NAC TFs has been proposed as important arbitrators of various plant processes and have been subjected to intensive investigations, especially in well-known model plants. As of now, no such study has been undertaken in *Setaria italica*, a model grass species in its class bestowed with potential caliber for stress-adaptation. Our data acquisition and systematic analysis has identified the entire *NAC* protein-encoding genes present in foxtail millet genome, for the first time. Variation in lengths and genomic structure were suggestive of the fact that, a great deal of complexity has evolved within this gene family. Phylogenetically, the NAC proteins belonged to eleven sub-families and had varied motif organization which seemed to be conserved for a particular sub-family. This analysis is an indication of functional conservation within a sub-family and serves as an initial platform in facilitating a better understanding of the structure-function relationship between individual members. *SiNAC* genes shared high orthology with their counter-parts in sorghum and maize supporting their close evolutionary relationship. A preliminary expression profiling of some *SiNAC* genes showed that their transcript accumulation were influenced by several environmental stimuli, including phytohormones and stress conditions, indicating their role in hormonal and stress response. We have also described the structure of a stress-responsive gene, SiNAC128. Despite these experiments, an arduous *in planta* characterization of each putative *SiNAC* gene is a prerequisite to explore their biological functioning. At this point of time, however, it is most prudent to suggest that this work enables us to propose a list of novel stress-responsive genes from foxtail millet which may be utilized as putative candidates by the research community to engineer improved adaptation capacity to agronomically important crops.

## Supporting Information

Figure S1 **Gene structures of 147** ***SiNAC*** **transcription factors.** Exons and introns are represented by green boxes and black lines, respectively. Scale represents the sizes of exons and introns can be estimated using the scale at bottom.
(TIF)

Figure S2 **Phylogenetic relationship of foxtail millet membrane-associate NAC transcription factors with those of Arabidopsis, rice and soybean.** Full-length amino acid sequences were aligned using ClustalW and the unrooted tree was constructed using MEGA5 by neighbor-joining method. The bootstrap values are shown at the nodes while the scale bar displays relative divergence among the sequences.
(TIF)

Figure S3 **Variation in motif clades for the SiNAC proteins.** The MEME motifs are shown as different-colored boxes at the N-terminal indicating the NAC domain region as well as the C-terminal region for the transcription regulatory region.
(TIF)

Figure S4 **Comparative physical mapping revealed high degree of orthologous relationships of NAC transcription factor genes located on nine chromosomes of foxtail millet with (A) rice, (B) maize and (C) sorghum.**
(TIF)

Figure S5 **Comparison of the secondary structures of SiNAC proteins belonging to different sub-families.** Key to figure: Blue line: Helix, Red Line: Strand, Pink Line: Coil, Green Line: Turn.
(TIF)

Figure S6 **Structure and molecular simulation analysis of SiNAC128.** (A) The original predicted structure of SiNAC128 prior to loop refinement as revealed by homology modeling. (B) Superimposed three-dimensional structures before and after molecular dynamic (MD) simulation. SiNAC128 structure after MD (C) within water molecules and (D) surrounded by ions.
(TIF)

Table S1 **List of primers used in quantitative real time-PCR expression analysis of 38 SiNAC genes.**
(DOC)

Table S2 **A catalog of** ***Setaria italica*** **NAC transcription factor proteins.**
(XLS)

Table S3 **The Ka/Ks ratios and estimated divergence time for orthologous NAC proteins between foxtail millet, rice, sorghum and maize.**
(DOC)

Table S4 **Fold expression values of 50 foxtail millet NAC genes in response to various stress and hormone treatments.**
(XLS)
