# Supplementary figures and images for: Comprehensive Genome-Wide Survey, Genomic Constitution and Expression Profiling of the NAC Transcription Factor Family in Foxtail Millet (Setaria italica L.)
Source: PLoS One. 2013 May 15;8(5):e64594. doi: 10.1371/journal.pone.0064594 (PMC3654982; doi:10.1371/journal.pone.0064594)

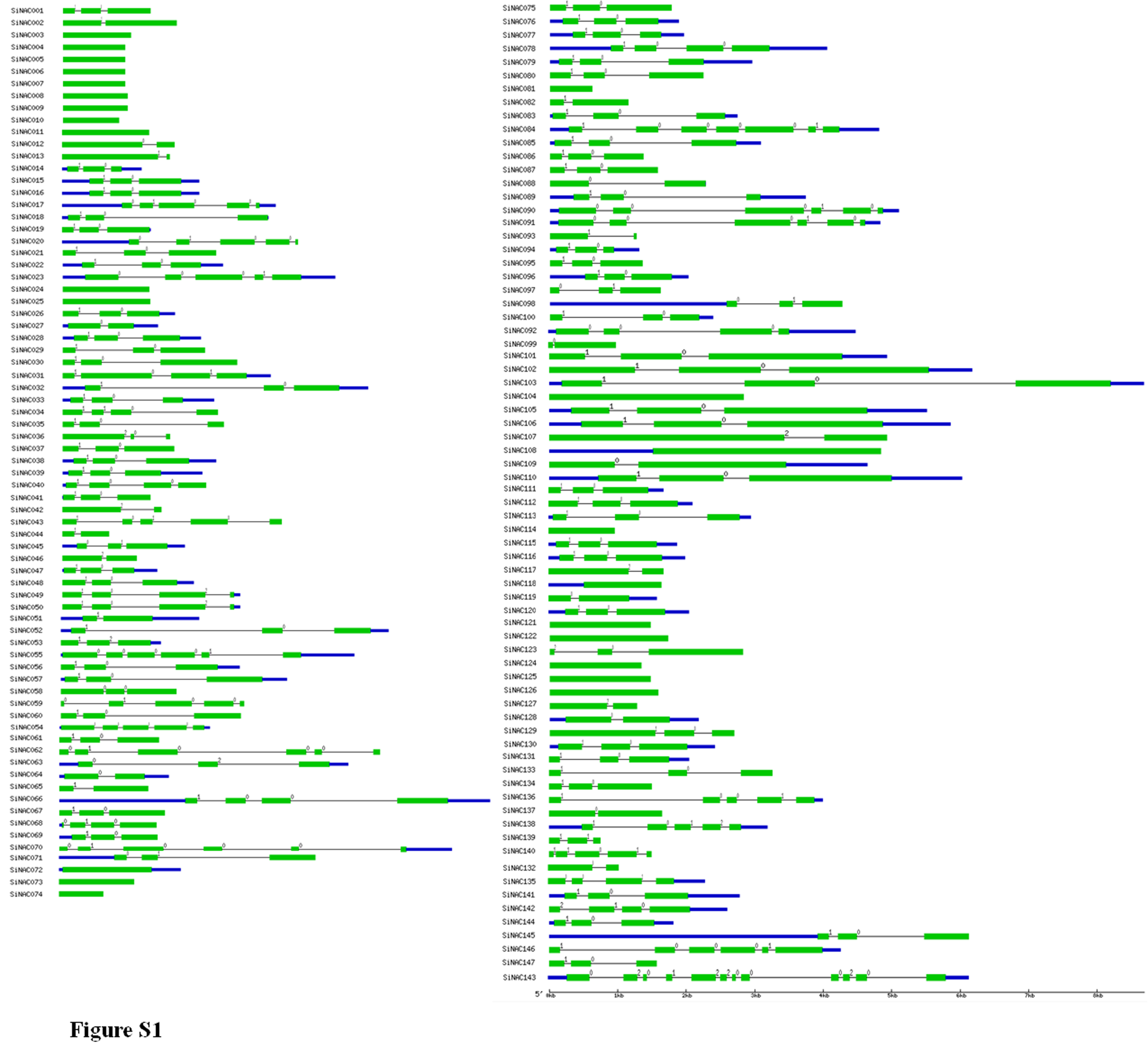

Supplement: Figure S1 — Gene structures of 147 SiNAC transcription factors. Exons and introns are represented by green boxes and black lines, respectively. Scale represents the sizes of exons and introns can be estimated using the scale at bottom. (TIF) [file pone.0064594.s001.tif]

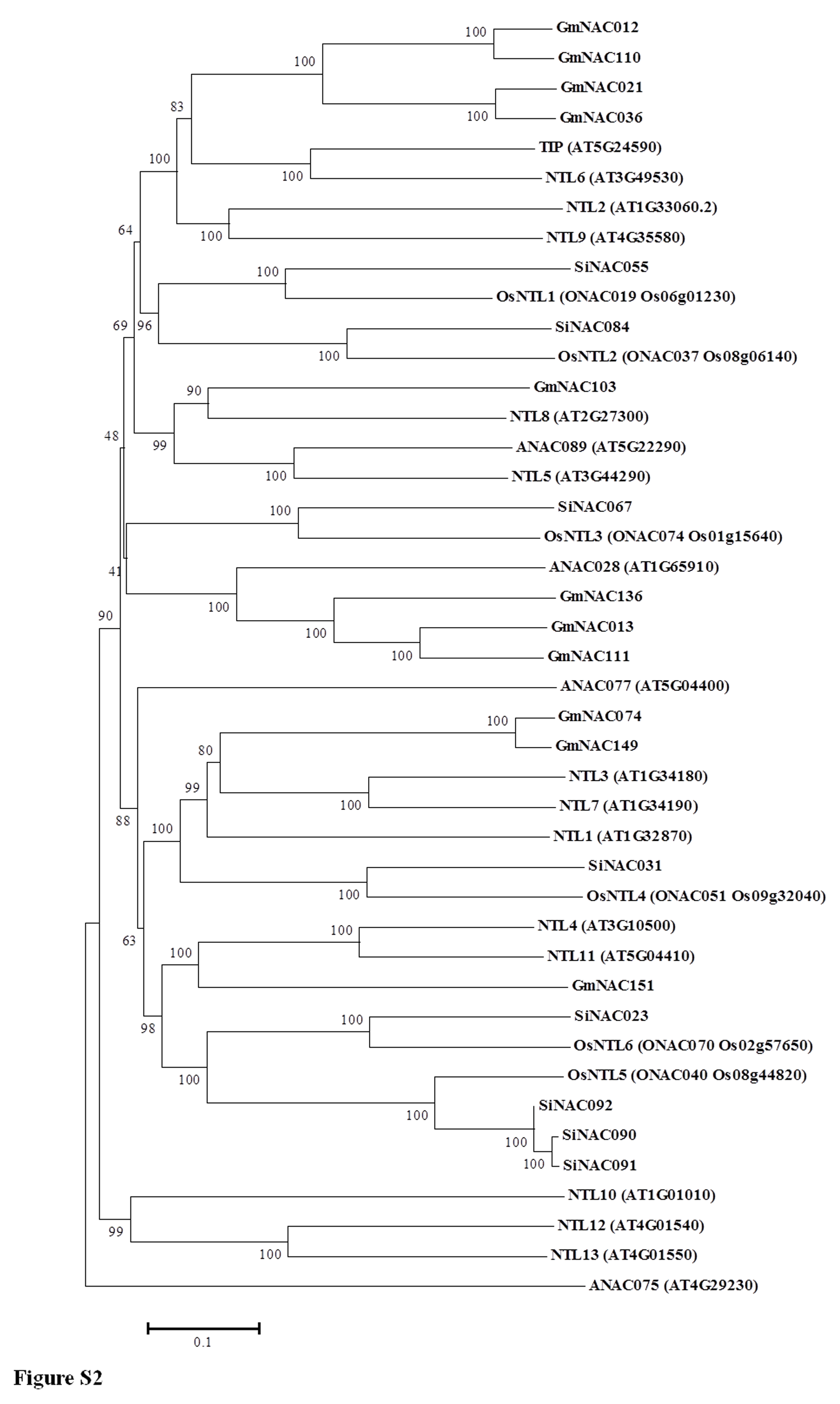

Supplement: Figure S2 — Phylogenetic relationship of foxtail millet membrane-associate NAC transcription factors with those of Arabidopsis, rice and soybean. Full-length amino acid sequences were aligned using ClustalW and the unrooted tree was constructed using MEGA5 by neighbor-joining method. The bootstrap values are shown at the nodes while the scale bar displays relative divergence among the sequences. (TIF) [file pone.0064594.s002.tif]

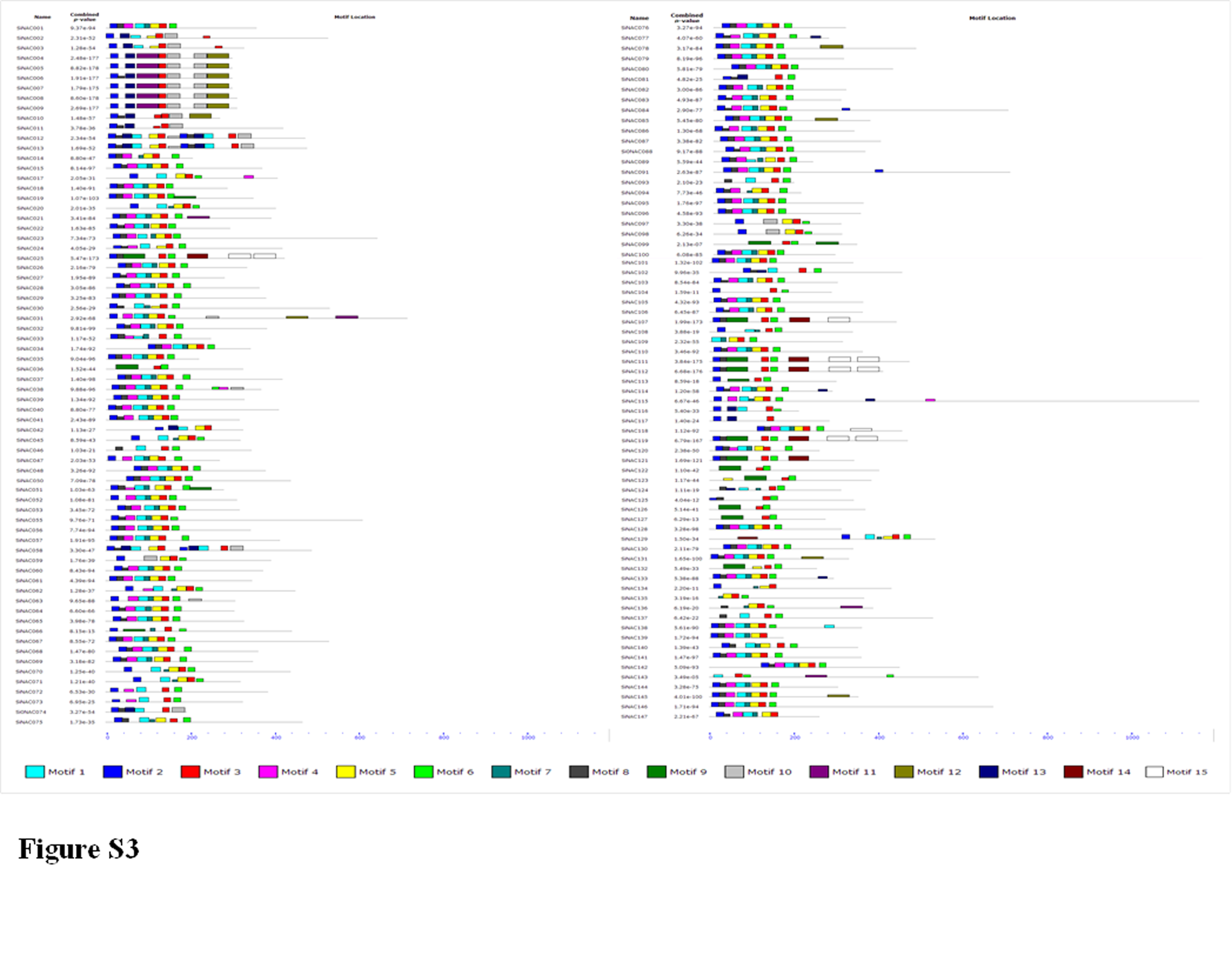

Supplement: Figure S3 — Variation in motif clades for the SiNAC proteins. The MEME motifs are shown as different-colored boxes at the N-terminal indicating the NAC domain region as well as the C-terminal region for the transcription regulatory region. (TIF) [file pone.0064594.s003.tif]

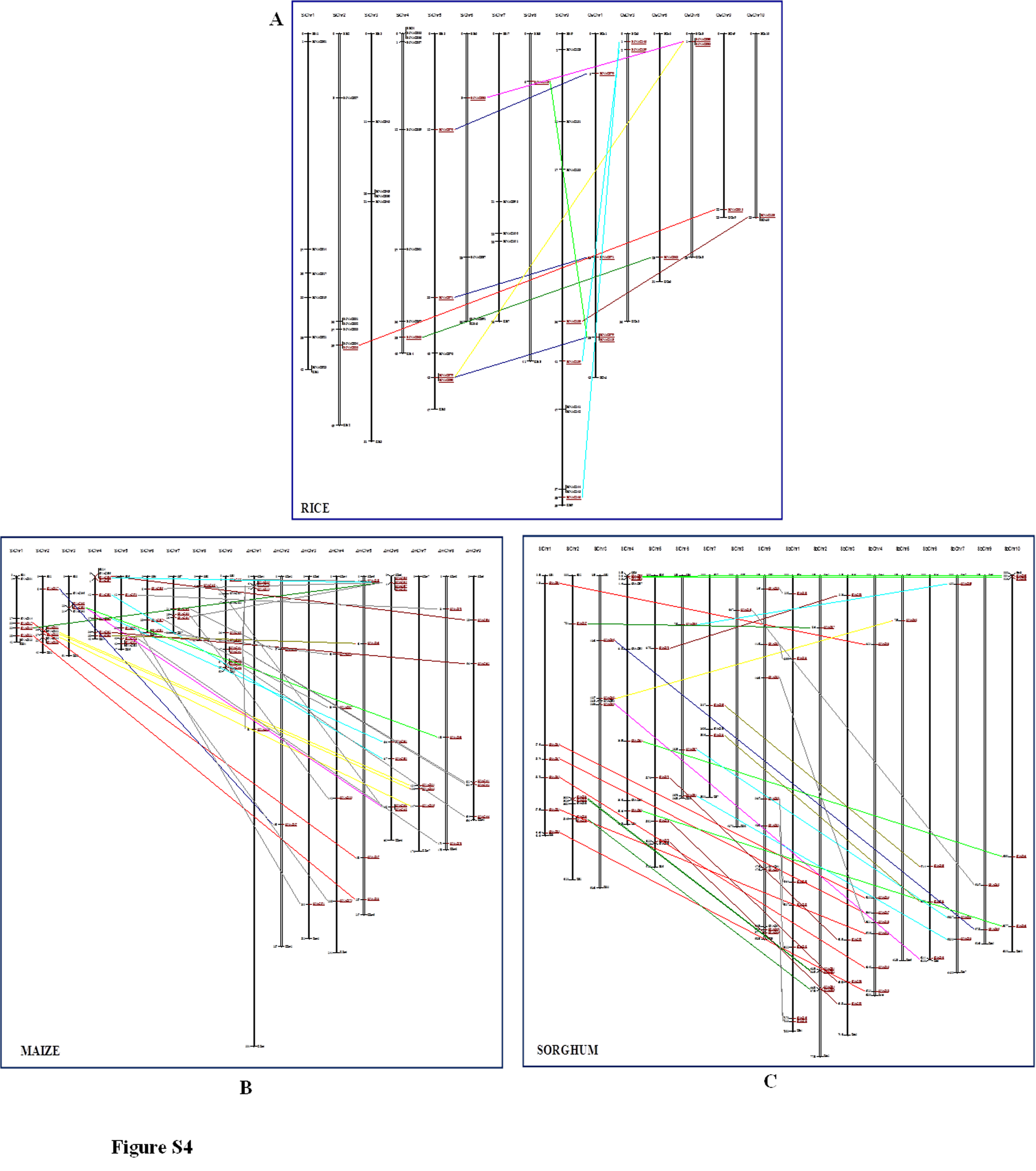

Supplement: Figure S4 — Comparative physical mapping revealed high degree of orthologous relationships of NAC transcription factor genes located on nine chromosomes of foxtail millet with (A) rice, (B) maize and (C) sorghum. (TIF) [file pone.0064594.s004.tif]

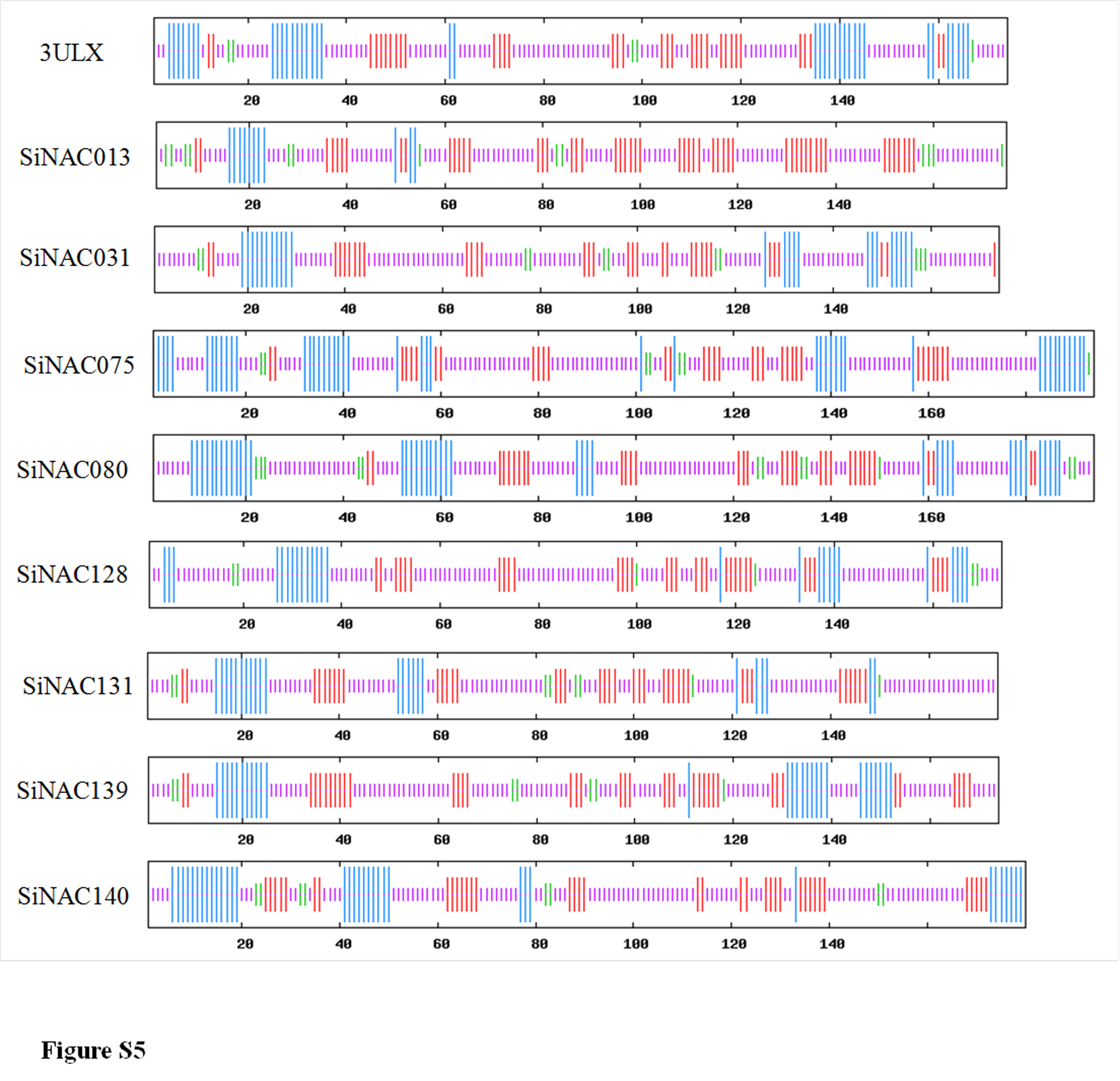

Supplement: Figure S5 — Comparison of the secondary structures of SiNAC proteins belonging to different sub-families. Key to figure: Blue line: Helix, Red Line: Strand, Pink Line: Coil, Green Line: Turn. (TIF) [file pone.0064594.s005.tif]

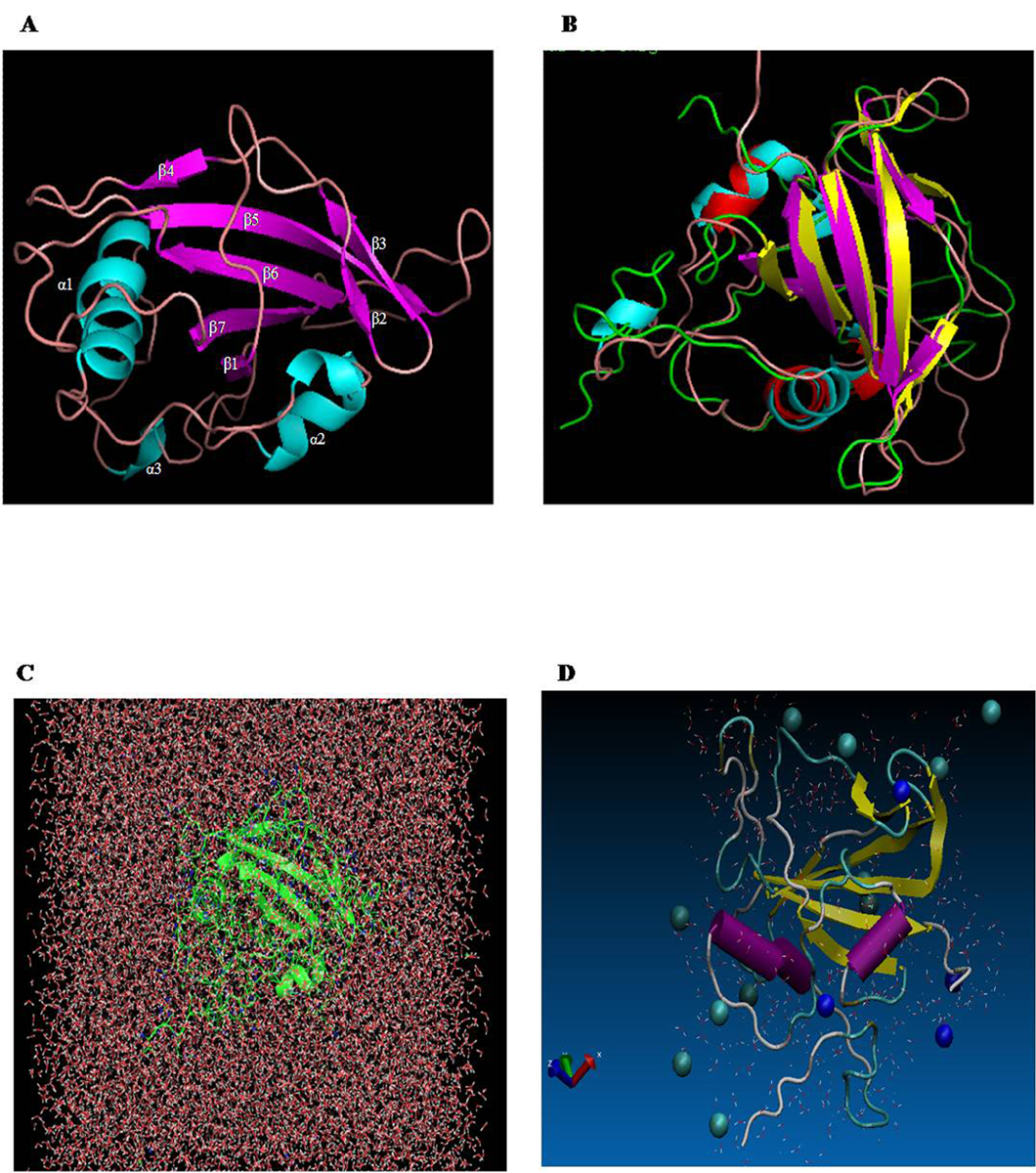

Supplement: Figure S6 — Structure and molecular simulation analysis of SiNAC128. (A) The original predicted structure of SiNAC128 prior to loop refinement as revealed by homology modeling. (B) Superimposed three-dimensional structures before and after molecular dynamic (MD) simulation. SiNAC128 structure after MD (C) within water molecules and (D) surrounded by ions. (TIF) [file pone.0064594.s006.tif]
