# Supplementary material for: Comprehensive Genome-Wide Survey, Genomic Constitution and Expression Profiling of the NAC Transcription Factor Family in Foxtail Millet (Setaria italica L.)
Source: PLoS One. 2013 May 15;8(5):e64594. doi: 10.1371/journal.pone.0064594 (PMC3654982; doi:10.1371/journal.pone.0064594)
Supplement: Table S1 — List of primers used in quantitative real time-PCR expression analysis of 38 SiNAC genes. (DOC) [file pone.0064594.s007.doc]

**Table S1.** List of primers used in quantitative real time-PCR expression analysis of 50 SiNAC genes.

| **NAME** | **FORWARD PRIMERS (5′- 3′)** | **REVERSE PRIMERS (5′- 3′)** |
| --- | --- | --- |
| SiNAC003 | ATCAGCGATTATCTGGGCTT | CTGCTGCTCCTCTGTACTCG |
| SiNAC014 | CACCTTCAAGATGGAGGATA | ATTCGAACACTCTGCATACC |
| SiNAC018 | GTGCAGGGTGTTTCAGAAGA | TAGTAGCAGTAGCCGCCTGA |
| SiNAC019 | AAGAGGCCGGTTACTCCAAT | TAGTCCAGTGCCTCTTGTGG |
| SiNAC024 | GGCGCTCATGAGGAAGTC | CAATTTGGAACCCGCACT |
| SiNAC027 | GATCAACCTCGACGACCT | TCAGAAGAACGGAAAGCC |
| SiNAC033 | CGTCGTCTTCAAGCTGTGTC | GGGGCATCTCTGATAGTGCT |
| SiNAC037 | ATGCAATCCCTACGTTCCTC | GGTAGGGATGCTGGAAACTG |
| SiNAC038 | AGATCTTCGACACCACCAC | GAGTAGATCGTCGTCAGGTC |
| SiNAC043 | CTCATGAGATTGGTGCAGTA | GACATGCTGTTGTGATGGTA |
| SiNAC045 | AGCTCACTTCATGCCTAACT | TCAAGGGCCAAAGGTAGC |
| SiNAC047 | GCCGGCTCTACACCAAGT | AGCCCTTCTCCTCTTCCG |
| SiNAC048 | AGAGCCAGCCCAAGATCG | GAGGATGTCCTGGAGCAG |
| SiNAC051 | GCGTTCATCGACTTCTTCG | GCAGCTCGACGACACCAC |
| SiNAC052 | TTCTCCAGCTTCAACAACAT | AGTTCAGAGGATCAAAGCAG |
| SiNAC055 | CAAACTTCACGGATCCACAC | CATCTTCACCCTCCGAATTT |
| SiNAC062 | ATTCCAGATCAGCACACCAA | CCATGGTCATACGTCTCCTG |
| SiNAC063 | CTCTCATCAGCCATCGTCAT | TCTTCACTACGTTGCCTTCG |
| SiNAC064 | AGAGACGACGACCATGATGA | ACTCGGTCACCACCTCCTT |
| SiNAC065 | GGCCATCACGACAACTACAG | GGAAGGACAGGTTCCTCTTG |
| SiNAC066 | CATGCCCATTACTACCACCA | CACGAGTAACCAGGCAACC |
| SiNAC070 | CCACGGCTTTCCTTATCAGT | GTAGAAGGATTGCTGGCACA |
| SiNAC071 | CACTGATCACCACCACCAC | ATGCCTGCTTCCTTTAGC |
| SiNAC073 | GGCTCACGGAGTACATGAAA | GGAGCCGAGGATCCATATC |
| SiNAC075 | CTGCCCGATGATGACTACC | ACGCTGAGCCTGTTAGTGC |
| SiNAC076 | GGTGAAGGAGGACAACGACT | TTAGGATTCACCAGCTGCAC |
| SiNAC078 | TGGTGAGGCTTGTGGAGTCA | CCCCAAGAGGGAAGAGATCAT |
| SiNAC079 | CTCGGAGCAGGTGCTGTC | CTCCCACTCGCTGATCTT |
| SiNAC083 | AGCTACCACCTGAAGATGCC | AAGGCGGTGTAGGTGTCAG |
| SiNAC086 | GTGTTCATGAGGAAGCAGCA | GTGTTCATGAGGAAGCAGCA |
| SiNAC088 | AAGTGGGTCCTCCACGAGTA | CAGATCACCCATTCCTCCTT |
| SiNAC089 | CTGGGTCATGCACGAGTA | ACACGACCCACTCTCCTT |
| SiNAC093 | TATTTCTCCTACAAGCGCAA | AACTCCACCATGTACCAGTC |
| SiNAC094 | TCAAGCAGGAAGTTGCACAG | CCCTCCTCGTGGTAGTTCAC |
| SiNAC096 | AGCCATCTCTCACCTCCCT | GTTGGAGAAGCAGGTCACG |
| SiNAC100 | TGATGTGGCAGTACAACTCG | GGAGCCTAGACCCATCCC |
| SiNAC101 | CGAAGGAATCCAGTTACTCCA | GGCCTCTTCTTCCTTCTCCT |
| SiNAC102 | GGGACCAGTGAAGATGCAG | AAGAGAGTTGGGAACGGTTG |
| SiNAC103 | GAGACGTCGTCACTGCTGTC | CGTCGTCATCTTGCTGGTAG |
| SiNAC105 | AGACGCATGTCCAGATTACG | CATCACTCGCTTCCTCTTCA |
| SiNAC108 | GCGTATACGTGAAGGACGAG | TCTAGACATTGCTGCCATCC |
| SiNAC110 | ATTACGTTGCGCCTAATAAC | CTTGGCAGCTTCAGTAATTT |
| SiNAC117 | GCAAGGAGAAGAAGAATGAG | GCTGCTGGTAGTAGTTGCTC |
| SiNAC118 | GATGAAGGTGGAGATGGC | GGTGTCTCGAGGTCGTAGT |
| SiNAC120 | CCCTTCTACAACTTCCTTGC | CTAACCTCCTCACCATCCTT |
| SiNAC125 | GAGTGCATTCACCGACGA | CCACGTACTCGTCATCCATC |
| SiNAC128 | GAACGAGTGGGAGAAGATGC | GAGTGGGAGGTGGTCATGT |
| SiNAC136 | TCAGCTAATGTGGTCCTTGG | GAAACCCTTGGCTTGAATGT |
| SiNAC141 | CTCTCCGAGCTCTTCAACG | GTGCTGGTGGTGATGGTG |
| SiNAC143 | AGGCACGATGGTCAACTACC | ATCTTCGCCTTCCAATCAAC |
| 18S-rRNA | CAATGGGAAGCAAGGCTGTAA | AACAATCCGAACTGAGGCAATC |
