# Supplementary material for: Comprehensive Genome-Wide Survey, Genomic Constitution and Expression Profiling of the NAC Transcription Factor Family in Foxtail Millet (Setaria italica L.)
Source: PLoS One. 2013 May 15;8(5):e64594. doi: 10.1371/journal.pone.0064594 (PMC3654982; doi:10.1371/journal.pone.0064594)
Supplement: Table S3 — The Ka/Ks ratios and estimated divergence time for orthologous NAC proteins between foxtail millet, rice, sorghum and maize. (DOC) [file pone.0064594.s009.doc]

**TABLE S3.** The Ka/Ks ratios and estimated divergence time for orthologous NAC proteins between foxtail millet, rice, sorghum and maize.

| **Foxtail-Rice** | |  |  | | **Ks** | **Ka** | **Ka/Ks** | **Mya** |
| --- | --- | --- | --- | --- | --- | --- | --- | --- |
| **NIPGR ID** | **Chrm** | **ID** | | **Chrm** |  |  |  |  |
| SiNAC035 | 2 | Os09g38010 | | 9 | 0.48 | 0.25 | 0.52 | 36.9 |
| SiNAC063 | 4 | Os06g46270 | | 6 | 0.46 | 0.26 | 0.57 | 35.4 |
| SiNAC070 | 5 | Os01g09550 | | 1 | 0.51 | 0.27 | 0.53 | 39.2 |
| SiNAC071 | 5 | Os01g48130 | | 1 | 0.48 | 0.27 | 0.56 | 36.9 |
| SiNAC079 | 5 | Os01g66120 | | 1 | 0.51 | 0.28 | 0.55 | 39.2 |
| SiNAC080 | 5 | Os08g02160 | | 8 | 0.5 | 0.28 | 0.56 | 38.5 |
| SiNAC085 | 6 | Os08g02160 | | 8 | 0.49 | 0.28 | 0.57 | 37.7 |
| SiNAC118 | 8 | Os01g66120 | | 1 | 0.46 | 0.27 | 0.59 | 35.4 |
| SiNAC138 | 9 | Os10g42130 | | 10 | 0.46 | 0.26 | 0.57 | 35.4 |
| SiNAC139 | 9 | Os03g03540 | | 3 | 0.47 | 0.28 | 0.60 | 36.2 |
| SiNAC146 | 9 | Os03g02800 | | 3 | 0.46 | 0.27 | 0.59 | 35.4 |
|  |  |  | | **Avg.** | **0.48** | **0.27** | **0.56** | **36.9** |
| **Foxtail-Sorghum** | |  |  | | **Ks** | **Ka** | **Ka/Ks** | **Mya** |
| **NIPGR ID** | **Chrm** | **ID** | | **Chrm** | 0.22 | 0.05 | 0.23 | 16.9 |
| SiNAC001 | 1 | Sb04g009180 | | 4 | 0.21 | 0.04 | 0.19 | 16.2 |
| SiNAC014 | 1 | Sb04g022672 | | 4 | 0.21 | 0.04 | 0.19 | 16.2 |
| SiNAC017 | 1 | Sb04g024780 | | 4 | 0.25 | 0.07 | 0.28 | 19.2 |
| SiNAC019 | 1 | Sb04g033570 | | 4 | 0.24 | 0.06 | 0.25 | 18.5 |
| SiNAC021 | 1 | Sb04g028015 | | 4 | 0.26 | 0.06 | 0.23 | 20.0 |
| SiNAC023 | 1 | Sb04g037720 | | 4 | 0.21 | 0.04 | 0.19 | 16.2 |
| SiNAC027 | 2 | Sb02g006680 | | 2 | 0.22 | 0.04 | 0.18 | 16.9 |
| SiNAC031 | 2 | Sb02g028750 | | 2 | 0.24 | 0.04 | 0.17 | 18.5 |
| SiNAC032 | 2 | Sb02g028870 | | 2 | 0.26 | 0.06 | 0.23 | 20.0 |
| SiNAC034 | 2 | Sb02g032220 | | 2 | 0.21 | 0.07 | 0.33 | 16.2 |
| SiNAC035 | 2 | Sb02g032230 | | 2 | 0.22 | 0.04 | 0.18 | 16.9 |
| SiNAC040 | 3 | Sb06g034210 | | 6 | 0.22 | 0.04 | 0.18 | 16.9 |
| SiNAC045 | 3 | Sb09g028430 | | 9 | 0.21 | 0.05 | 0.24 | 16.2 |
| SiNAC048 | 3 | Sb05g005450 | | 5 | 0.25 | 0.06 | 0.24 | 19.2 |
| SiNAC055 | 4 | Sb10g000101 | | 10 | 0.24 | 0.05 | 0.21 | 18.5 |
| SiNAC056 | 4 | Sb10g000460 | | 10 | 0.26 | 0.05 | 0.19 | 20.0 |
| SiNAC061 | 4 | Sb10g020860 | | 10 | 0.26 | 0.05 | 0.19 | 20.0 |
| SiNAC063 | 4 | Sb10g027100 | | 10 | 0.22 | 0.07 | 0.32 | 16.9 |
| SiNAC070 | 5 | Sb03g003170 | | 3 | 0.21 | 0.06 | 0.29 | 16.2 |
| SiNAC071 | 5 | Sb03g030750 | | 3 | 0.21 | 0.06 | 0.29 | 16.2 |
| SiNAC076 | 5 | Sb03g037940 | | 3 | 0.23 | 0.04 | 0.17 | 17.7 |
| SiNAC079 | 5 | Sb03g041920 | | 3 | 0.24 | 0.04 | 0.17 | 18.5 |
| SiNAC085 | 6 | Sb07g001400 | | 7 | 0.25 | 0.04 | 0.16 | 19.2 |
| SiNAC087 | 6 | Sb07g021400 | | 7 | 0.22 | 0.06 | 0.27 | 16.9 |
| SiNAC091 | 6 | Sb07g023900 | | 7 | 0.24 | 0.07 | 0.29 | 18.5 |
| SiNAC095 | 7 | Sb06g017720 | | 6 | 0.24 | 0.07 | 0.29 | 18.5 |
| SiNAC101 | 7 | Sb06g023780 | | 6 | 0.23 | 0.06 | 0.26 | 17.7 |
| SiNAC118 | 8 | Sb09g020750 | | 9 | 0.25 | 0.04 | 0.16 | 19.2 |
| SiNAC128 | 9 | Sb01g003710 | | 1 | 0.26 | 0.07 | 0.27 | 20.0 |
| SiNAC131 | 9 | Sb01g014310 | | 1 | 0.23 | 0.04 | 0.17 | 17.7 |
| SiNAC133 | 9 | Sb04g026440 | | 4 | 0.25 | 0.06 | 0.24 | 19.2 |
| SiNAC138 | 9 | Sb01g028450 | | 1 | 0.23 | 0.06 | 0.26 | 17.7 |
| SiNAC139 | 9 | Sb01g030760 | | 1 | 0.21 | 0.05 | 0.24 | 16.2 |
| SiNAC142 | 9 | Sb01g036590 | | 1 | 0.21 | 0.05 | 0.24 | 16.2 |
| SiNAC145 | 9 | Sb01g048730 | | 1 | 0.23 | 0.04 | 0.17 | 17.7 |
| SiNAC146 | 9 | Sb01g049240 | | 1 | 0.21 | 0.04 | 0.19 | 16.2 |
|  |  |  | | **Avg.** | **0.23** | **0.05** | **0.22** | **17.7** |
| **Foxtail-Maize** | |  |  | | **Ks** | **Ka** | **Ka/Ks** | **Mya** |
| **NIPGR ID** | **Chrm** | **ID** | | **Chrm** |  |  |  |  |
| SiNAC017 | 1 | GRMZM2G038073 | | 5 | 0.26 | 0.06 | 0.23 | 20.0 |
| SiNAC019 | 1 | GRMZM2G092465 | | 6 | 0.28 | 0.07 | 0.25 | 21.5 |
| SiNAC021 | 1 | GRMZM2G100593 | | 5 | 0.29 | 0.08 | 0.28 | 22.3 |
| SiNAC027 | 2 | GRMZM2G162739 | | 2 | 0.29 | 0.09 | 0.31 | 22.3 |
| SiNAC031 | 2 | GRMZM2G004531 | | 7 | 0.27 | 0.09 | 0.33 | 20.8 |
| SiNAC033 | 2 | GRMZM2G054277 | | 7 | 0.26 | 0.07 | 0.27 | 20.0 |
| SiNAC035 | 2 | GRMZM5G885329 | | 7 | 0.26 | 0.07 | 0.27 | 20.0 |
| SiNAC048 | 3 | GRMZM2G180328 | | 6 | 0.28 | 0.07 | 0.25 | 21.5 |
| SiNAC050 | 3 | GRMZM2G134687 | | 8 | 0.29 | 0.06 | 0.21 | 22.3 |
| SiNAC056 | 4 | GRMZM2G440219 | | 9 | 0.24 | 0.08 | 0.33 | 18.5 |
| SiNAC057 | 4 | GRMZM2G092465 | | 6 | 0.24 | 0.09 | 0.38 | 18.5 |
| SiNAC059 | 4 | GRMZM2G078954 | | 6 | 0.25 | 0.07 | 0.28 | 19.2 |
| SiNAC061 | 4 | GRMZM2G041746 | | 6 | 0.26 | 0.07 | 0.27 | 20.0 |
| SiNAC062 | 4 | GRMZM2G115721 | | 9 | 0.24 | 0.09 | 0.38 | 18.5 |
| SiNAC063 | 4 | GRMZM2G063522 | | 5 | 0.24 | 0.09 | 0.38 | 18.5 |
| SiNAC070 | 5 | GRMZM2G112681 | | 8 | 0.29 | 0.09 | 0.31 | 22.3 |
| SiNAC071 | 5 | GRMZM2G058518 | | 3 | 0.28 | 0.07 | 0.25 | 21.5 |
| SiNAC076 | 5 | GRMZM2G068973 | | 8 | 0.27 | 0.09 | 0.33 | 20.8 |
| SiNAC079 | 5 | GRMZM2G123667 | | 4 | 0.27 | 0.08 | 0.30 | 20.8 |
| SiNAC085 | 6 | GRMZM2G030325 | | 6 | 0.25 | 0.08 | 0.32 | 19.2 |
| SiNAC087 | 6 | AC198937.4_FG005 | | 4 | 0.24 | 0.09 | 0.38 | 18.5 |
| SiNAC091 | 6 | GRMZM2G125777 | | 4 | 0.24 | 0.08 | 0.33 | 18.5 |
| SiNAC095 | 7 | GRMZM2G099144 | | 2 | 0.26 | 0.07 | 0.27 | 20.0 |
| SiNAC100 | 7 | GRMZM2G062009 | | 4 | 0.27 | 0.08 | 0.30 | 20.8 |
| SiNAC101 | 7 | GRMZM2G092465 | | 6 | 0.28 | 0.09 | 0.32 | 21.5 |
| SiNAC118 | 8 | GRMZM2G180328 | | 6 | 0.29 | 0.08 | 0.28 | 22.3 |
| SiNAC128 | 9 | GRMZM2G018553 | | 5 | 0.24 | 0.07 | 0.29 | 18.5 |
| SiNAC138 | 9 | GRMZM2G082709 | | 1 | 0.25 | 0.07 | 0.28 | 19.2 |
| SiNAC139 | 9 | GRMZM2G025642 | | 1 | 0.24 | 0.08 | 0.33 | 18.5 |
| SiNAC141 | 9 | GRMZM2G042494 | | 9 | 0.26 | 0.09 | 0.35 | 20.0 |
| SiNAC142 | 9 | GRMZM2G159500 | | 9 | 0.26 | 0.08 | 0.31 | 20.0 |
| SiNAC144 | 9 | GRMZM2G059428 | | 1 | 0.27 | 0.08 | 0.30 | 20.8 |
| SiNAC145 | 9 | GRMZM2G025642 | | 1 | 0.24 | 0.09 | 0.38 | 18.5 |
| SiNAC146 | 9 | GRMZM2G174070 | | 9 | 0.24 | 0.08 | 0.33 | 18.5 |
|  |  |  | | **Avg.** | **0.26** | **0.08** | **0.31** | **20.0** |
